# Entrenamiento de la Marcha Mediante Dispositivos Robóticos en Pacientes con Ictus: Una Revisión de Revisiones Sistemáticas y Metaanálisis

**DOI:** 10.31083/RN46880

**Published:** 2026-03-18

**Authors:** Juan Antonio Chamorro-Hinojosa, Francisco Molina-Rueda, María Carratalá-Tejada

**Affiliations:** ^1^Escuela Internacional de Doctorado, Universidad Rey Juan Carlos. Rectorado – Delegación Madrid, 28008 Madrid, España; ^2^Departamento de Fisioterapia, Terapia Ocupacional, Rehabilitación y Medicina Física, Facultad de Ciencias de la Salud, Universidad Rey Juan Carlos, 28922 Alcorcón, Madrid, España

**Keywords:** fisioterapia, neurorrehabilitación, dispositivos robóticos, ictus, marcha, physical therapy modalities, rehabilitation, robotic devices, stroke, gait

## Abstract

**Introducción:**

El entrenamiento de la marcha mediante dispositivos robóticos en pacientes con ictus constituye una modalidad de tratamiento ampliamente investigada. Por tanto, existe mucha información heterogénea que es necesario sintetizar, ordenar y clasificar. Por ello, el objetivo de este trabajo es sintetizar y analizar la evidencia científica sobre la aplicación de dispositivos robóticos para el entrenamiento de la marcha en personas con ictus.

**Métodos:**

Esta revisión de revisiones sistemáticas y metaanálisis se llevó a cabo siguiendo las recomendaciones de *Preferred Reporting Items for Systematic Reviews and Meta-Analyses* (PRISMA). Se realizaron búsquedas en 4 bases de datos electrónicas: PubMed, Scopus, Web of Science y Cochrane Library Plus. Se incluyeron revisiones sistemáticas y metaanálisis que incluyeran ensayos controlados aleatorizados (ECA) que investigasen los efectos de los dispositivos robóticos en combinación o no con otro tratamiento de fisioterapia en la recuperación de la marcha en pacientes con ictus.

**Resultados:**

Se incluyeron 13 estudios con un total de 101 ECA. Se extrajeron los datos relativos a la población, medidas de resultados, los protocolos de entrenamiento y los resultados principales. Se aplicó la escala *A Messurement Tool to Assess Systematic Review* (AMSTAR-2) y el Sistema *Grading of Recommendations Assessment, Development, and Evaluation* (GRADE) de certeza de la evidencia. Solo un estudio tuvo una certeza de evidencia alta; mientras que, cuatro obtuvieron una certeza moderada, seis se clasificaron con una certeza baja y dos obtuvieron una calidad críticamente baja.

**Conclusiones:**

El entrenamiento robótico combinado con fisioterapia mejora la velocidad de marcha tras un ictus, especialmente con efectores finales. Sin embargo, los beneficios no alcanzan límites funcionales clínicamente significativos y su aplicabilidad es limitada por la evidencia, el coste y la accesibilidad.

**El número de registro de PROSPERO:**

CRD42021237915, https://www.crd.york.ac.uk/PROSPERO/view/CRD42021237915.

## Abstract (en)

**Introduction:**

Gait training using robotic devices in stroke patients is a widely researched treatment modality. Therefore, there is a lot of heterogeneous information that needs to be synthesized, sorted, and classified. The aim of this work was to synthesize and analyze the scientific evidence on the application of robotic devices for gait training in people with stroke.

**Methods:**

This overview of systematic reviews and meta-analysis was carried out following the Preferred Reporting Items for Systematic Reviews and Meta-Analyses (PRISMA) recommendations. Searches were performed in four electronic databases: PubMed, Scopus, Web of Science, and Cochrane Library Plus. Systematic reviews and meta-analyses that included randomized controlled trials (RCTs) that investigated the effects of robotic devices in combination or not with another physiotherapy treatment on gait recovery in stroke patients were included.

**Results:**

Thirteen studies with a total of 101 RCTs were included. Data regarding the participants, outcome measures, training protocols and main results were extracted. The A Messurement Tool to Assess Systematic Review (AMSTAR-2) scale and the Grading of Recommendations Assessment, Development, and Evaluation (GRADE) system of certainty of evidence were applied. Only one study had a high certainty of evidence; while four had a moderate certainty, six were classified as having a low certainty and two had a critically low quality.

**Conclusions:**

Robotic gait training combined with physiotherapy improves walking speed after stroke, especially with end-effector devices. However, benefits do not reach clinically meaningful functional thresholds, and applicability is limited due to insufficient evidence, high costs, and limited accessibility.

## 1. Introducción

El accidente cerebrovascular (ACV) es una de las principales causas de discapacidad en todo el mundo, afectando la movilidad y la marcha de los supervivientes [1]. La alteración en el patrón de marcha impacta en las actividades de la vida diaria y aumenta el riesgo de caídas, lo que deteriora la calidad de vida. Se estima que hasta el 50% de los pacientes con ictus experimentarán caídas en el primer año tras el evento [2], lo que hace de la mejora de la marcha un objetivo fundamental en la rehabilitación post-ictus [3, 4].

En los últimos años, se ha incrementado el uso de nuevas tecnologías en la neurorrehabilitación, especialmente con dispositivos robóticos diseñados para mejorar la recuperación funcional. Estas tecnologías representan un avance respecto a los métodos tradicionales y han generado expectativas, aunque también plantean retos para los profesionales sanitarios encargados de evaluar su efectividad [5, 6]. En este contexto, aunque enfoques como la electroestimulación, la realidad virtual y la biorretroalimentación han mostrado beneficios, su nivel de evidencia sigue siendo limitado según la American Stroke Association, lo que reduce la recomendación para su uso en la rehabilitación [7].

El entrenamiento de la marcha mediante dispositivos robóticos se basa en una serie de principios fundamentales que, a priori, suponen una ventaja respecto a los procedimientos convencionales de neurorrehabilitación. En primer lugar, la alta repetición de la tarea permite aplicar tratamientos más intensivos. En segundo lugar, la asistencia o facilitación proporcionada por estos dispositivos favorece la participación del paciente en sus actividades cotidianas. En tercer lugar, la estimulación multisensorial integrada en el entrenamiento permite combinar información visual, propioceptiva y táctil, optimizando el proceso de aprendizaje. Por último, la monitorización continua que ofrecen los sistemas robóticos posibilita adaptar parámetros de la marcha —como la velocidad o la resistencia— y proporcionar retroalimentación en tiempo real tanto al paciente como a los profesionales, lo que repercute positivamente en la motivación y en el aprendizaje motor adquirido [8, 9, 10]. Estas son características fundamentales para promover el aprendizaje motor y la neuroplasticidad y de esta manera, mejorar la funcionalidad de los pacientes [11, 12].

Respecto a los dispositivos robóticos empleados para el entrenamiento de la marcha, existen diferentes modalidades. Por un lado, hay dispositivos fijos, que suelen integrar sistemas de suspensión del peso corporal y entrenar la marcha sobre cintas sin fin; y, por otro lado, existen dispositivos que son portables, permitiendo al usuario caminar en distintos terrenos. Además, de acuerdo con la interacción del dispositivo con el cuerpo del paciente, estos dispositivos pueden actuar como exoesqueletos que recubren los segmentos corporales, y otros, los denominados efectores finales, que asisten a la marcha sobre cintas sin fin mediante una solución mecánica que interacciona con los segmentos distales de las extremidades inferiores del individuo [10].

A pesar de la evidencia que respalda los beneficios de estos dispositivos, su implementación presenta desafíos [13, 14, 15, 16]. La variedad de dispositivos disponibles y la heterogeneidad en los protocolos de intervención dificultan la comparación de resultados y su extrapolación a diferentes pacientes. Además, el coste-efectividad y la accesibilidad de estos dispositivos siguen siendo factores importantes que considerar en su aplicación.

Esta cuestión puede abordarse mediante revisiones sistemáticas, las cuales sintetizan la evidencia procedente de múltiples estudios sobre intervenciones dirigidas a un mismo problema de salud, facilitando así la toma de decisiones basada en la evidencia. Estas revisiones comparan dos o más intervenciones y, para su publicación, deben cumplir estrictos criterios de rigor metodológico y relevancia clínica [17]. En este contexto, el objetivo del presente estudio es realizar una revisión de revisiones sistemáticas y metaanálisis que hayan analizado el impacto de los dispositivos robóticos en la recuperación de la marcha en pacientes con ictus. Este análisis incluye la elaboración de tablas de evidencia estructuradas que permitan formular recomendaciones clínicas sobre las distintas aplicaciones de esta tecnología. Para ello, se empleará el sistema GRADE [18], que facilita la valoración de la calidad de la evidencia y orienta la práctica clínica a partir de datos objetivos y comparables.

## 2. Materiales y Métodos

### 2.1 Estrategia de Búsqueda en Base de Datos

Esta revisión se llevó a cabo siguiendo las directrices de la declaración Preferred Reporting Items for Systematic Reviews and Meta-Analyses (PRISMA) [19]. El PRISMA Checklist se incluye en el **Material Suplementario**. Se realizó una búsqueda en PubMed (https://pubmed.ncbi.nlm.nih.gov/), Scopus (https://www.scopus.com/pages/home), Web of Science (https://www.webofscience.com/) y Cochrane Database of Systematic Reviews (https://www.cochranelibrary.com/) el 21 de marzo de 2023, utilizando términos como “*stroke*”, “*cerebrovascular accident*”, “*apraxia of gait*” y “*gait dysfunction*”, combinados con operadores booleanos *AND* y *OR* (**Suplementario Tabla 1**). No se incluyó literatura gris para garantizar la revisión por pares, excluyendo tesis no publicadas, resúmenes de conferencias e informes no revisados. No se consultó a expertos ni se consideraron disertaciones académicas. La revisión fue inscrita en PROSPERO (CRD42021237915).

### 2.2 Criterios de Elegibilidad

- Tipo de estudios: se incluyeron revisiones sistemáticas (con o sin metaanálisis) que analizasen ensayos controlados aleatorizados para su revisión. Publicados desde el año 2015 hasta 2023. Se consideraron solo los títulos escritos en inglés y español.
- Tipo de participantes: Se consideraron revisiones que incluyesen: sujetos diagnosticados de ACV, mayores de 18 años, con ictus de tipo isquémico o hemorrágico en fase aguda, subaguda o crónica.
- Tipo de intervención: se consideraron trabajos que evaluasen algún tipo de dispositivo robótico para el entrenamiento de la marcha en combinación o no con otra forma de tratamiento fisioterápico. No se consideraron aquellos trabajos que no definieron las intervenciones o que evaluasen múltiples terapias y también cualquier tratamiento farmacológico (ej. toxina botulínica).
- Tipo de medidas de resultado: estudios que incluyan escalas o test de evaluación instrumental de la marcha humana, de análisis de la velocidad de marcha, de la resistencia de marcha o pruebas de movilidad funcional y/o funcionamiento, función motora gruesa y fuerza muscular.

### 2.3 Selección de Estudios y Extracción de Datos

La compilación de información se basó en el manual Cochrane de revisiones sistemáticas de intervenciones [17]. Dos autores evaluaron independientemente la inclusión de todas las revisiones sistemáticas identificadas en la búsqueda, resolviendo las discrepancias mediante discusión o consulta con un tercer miembro del equipo. Posteriormente, dos autores extrajeron de manera independiente los datos mediante una forma predefinida que incluía características de las revisiones y resúmenes estadísticos. En caso de información incompleta, se contactó a los autores por correo electrónico. Además, se calculó el *Corrected Covered Area* (CCA) [20] para cuantificar el solapamiento de estudios primarios entre revisiones y orientar la selección y priorización de la evidencia más reciente, completa y de mayor calidad para cada resultado.

### 2.4 Evaluación Metodológica y de la Calidad de la Evidencia

La calidad metodológica de las revisiones se evaluó con AMSTAR-2 (*A Messurement Tool to Assess Systematic Review*) [21], que clasifica la confianza en cuatro niveles: alta, media, baja y críticamente baja.

La calidad de la evidencia sobre la aplicación de los dispositivos robóticos para el entrenamiento de la marcha en pacientes con ictus se valoró con el instrumento *Grading of Recommendations Assessment, Development, and Evaluation* (GRADE) [18], utilizando GRADEpro GDT (https://gradepro.org/, McMaster University and Evidence Prime Inc., Hamilton, ON, Canada) [22] y siguiendo las directrices del manual Cochrane.

Todas las decisiones sobre la clasificación de la evidencia fueron justificadas para garantizar la transparencia del proceso de recomendación.

### 2.5 De la Evidencia a la Recomendación Clínica

Tras completar la revisión, se realizó una síntesis crítica de la evidencia disponible para identificar la revisión sistemática o metaanálisis más representativo.

La selección se basó en criterios como actualidad, número y representatividad de los estudios incluidos, calidad metodológica, limitaciones, resultados estadísticos y certeza de la evidencia según GRADE. Además, el solapamiento de los estudios primarios se evaluó manualmente, extrayéndose y organizándose esta información en una tabla [18].

## 3. Resultados

**Fig. 1.**
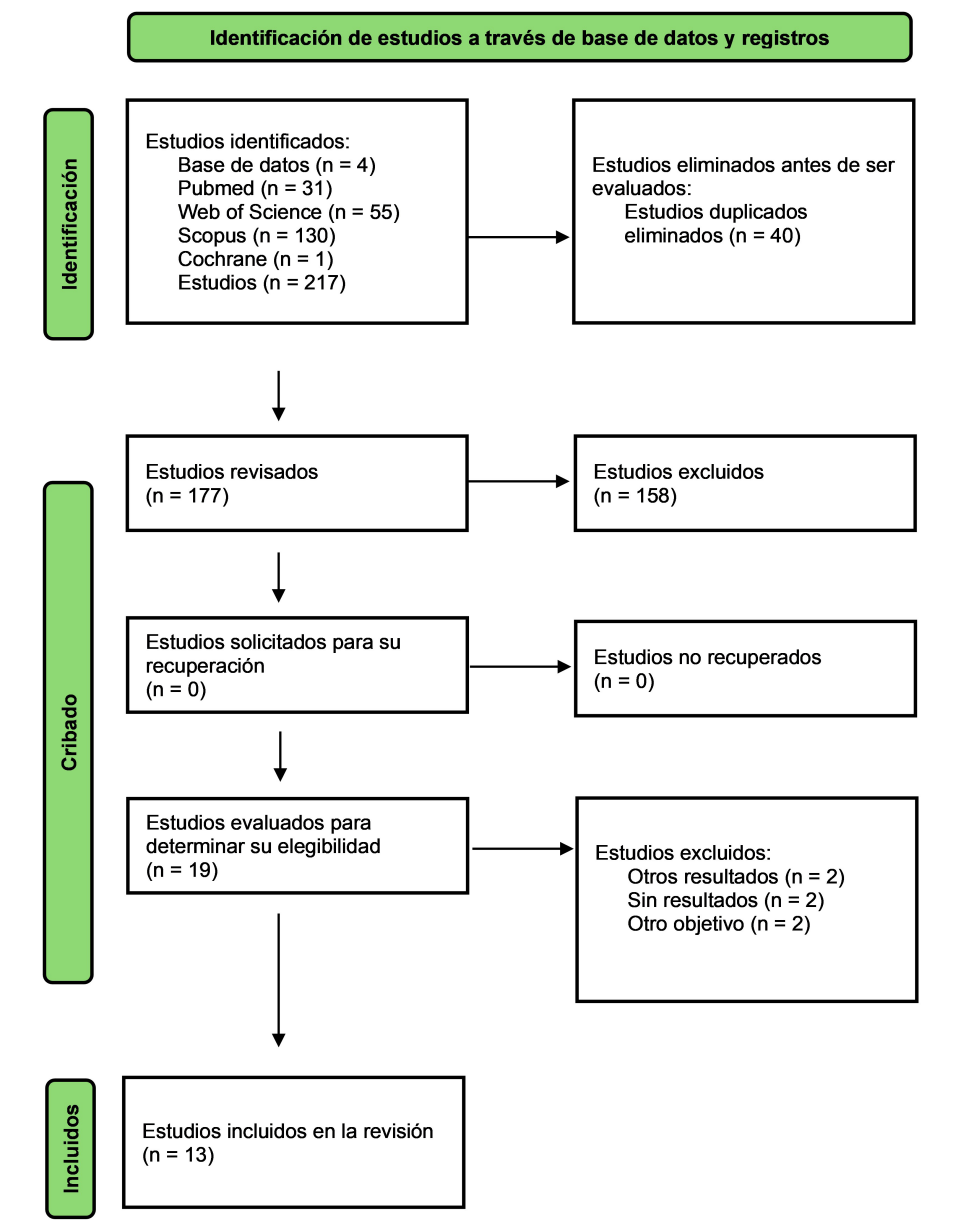
**Diagrama de flujo de selección de revisiones sistemáticas y metaanálisis sobre dispositivos robóticos para el entrenamiento de la marcha en ictus**.

Se encontraron un total de 217 estudios, se eliminaron los duplicados quedando un total de 177 artículos. Se realizó un cribado de los estudios por título y por resumen. Finalmente, se incluyeron 13 trabajos [13, 14, 15, 16, 23, 24, 25, 26, 27, 28, 29, 30, 31, 32, 33, 34] con un total de 101 estudios controlados aleatorizados (ECA). El proceso de selección se muestra en el diagrama de flujo (Fig. 1) con una lista de los estudios excluidos y sus razones (**Suplementario Tabla 2**). Paralelamente, se evaluó el solapamiento de los estudios primarios para evitar la doble contabilización de los datos mediante el método del CCA, construyéndose una matriz de citas. El análisis de las 13 revisiones sistemáticas mostró un CCA de 0,085, lo que, según los criterios de Pieper *et al*. [20], corresponde a un nivel de solapamiento moderado.

Las características de las revisiones y los resultados de la evaluación de la calidad metodológica se muestran en las Tabla 1 (Ref. [13, 14, 15, 16, 23, 26, 27, 28, 30, 31, 32, 33, 34]) y Tabla 2 (Ref. [13, 14, 15, 16, 23, 26, 27, 28, 30, 31, 32, 33, 34]); mientras que, los estudios incluidos en cada una de las revisiones, y la certeza de la evidencia para los desenlaces se presenta en el **Material Suplementario** (**Suplementario Tablas 3,4**). Los estudios revisados abarcan pacientes con ictus en distintas fases de recuperación, desde aguda/subaguda hasta crónica. La edad media varía entre 40 y 76 años, con predominio masculino. El tamaño muestral oscila entre 300 y más de 1500 participantes. La mayoría incluye pacientes con ictus isquémico. Todos los estudios incluyeron como forma de tratamiento un dispositivo robótico de entrenamiento de la marcha, clasificándose los dispositivos de la siguiente manera: (1) Exoesqueleto no portable: Lokomat® y Walkbot® [13, 14, 15, 16, 26, 31, 34]. (2) Dispositivo de efector final: G-EO® [13, 31, 33, 34]. (3) Exoesqueleto portátil: HAL®, Tibion Bionic Leg® y Ekso GT® [27]. (4) Dispositivos robóticos combinados [28, 30, 31, 32, 34].

**Tabla 1.** **Puntuación de la escala AMSTAR-2 de las revisiones sistemáticas y metaanálisis incluidos sobre el uso de dispositivos robóticos para el entrenamiento de la marcha en el ictus**.

AMSTAR	Cho *et al*. (2018) [15]	Bruni *et al*. (2018) [13]	Postol *et al*. (2019) [14]	Tedla *et al*. (2019) [16]	Maranesi *et al*. (2020) [33]	Schröder *et al*. (2019) [31]	Mehrholz *et al*. (2020) [34]	Moucheboeuf *et al*. (2020) [32]	Hsu *et al*. (2020) [30]	Nedergård *et al*. (2021) [28]	Calafiore *et al*. (2022) [26]	Hsu *et al*. (2023) [27]	Zhang *et al*. (2023) [23]
1	Y	Y	Y	Y	Y	Y	Y	Y	Y	Y	Y	Y	Y
2	PY	PY	Y	PY	PY	PY	Y	Y	PY	Y	Y	Y	Y
3	N	N	N	N	N	N	N	N	N	N	N	N	N
4	PY	PY	Y	PY	PY	PY	Y	Y	PY	PY	PY	PY	Y
5	Y	N	Y	Y	Y	Y	Y	Y	Y	Y	Y	Y	Y
6	Y	N	Y	Y	Y	Y	Y	Y	Y	Y	Y	Y	Y
7	PY	N	PY	PY	PY	PY	Y	PY	PY	PY	PY	PY	PY
8	Y	N	PY	PY	Y	Y	Y	Y	Y	Y	Y	Y	Y
9	Y	N	Y	Y	Y	Y	Y	Y	Y	Y	Y	Y	Y
10	N	N	N	N	N	N	Y	N	N	N	N	N	N
11	NA	Y	Y	N	NA	Y	Y	Y	Y	Y	Y	Y	Y
12	NA	Y	Y	N	NA	Y	Y	Y	Y	Y	Y	Y	Y
13	Y	Y	Y	Y	Y	Y	Y	Y	Y	Y	Y	Y	Y
14	N	N	Y	Y	Y	Y	Y	Y	Y	Y	Y	Y	Y
15	NA	N	N	N	NA	N	Y	Y	N	N	N	N	Y
16	Y	Y	N	Y	Y	Y	Y	Y	Y	Y	Y	Y	Y
Evaluación general	Moderada	Críticamente baja	Baja	Críticamente baja	Moderada	Baja	Alta	Moderada	Baja	Baja	Baja	Baja	Moderada

1, Pregunta PICO (Paciente, Intervención, Comparación y Outcome); 2, Protocolo registrado antes de la revisión; 3, Justificación del diseño de los estudios incluidos; 4, Adecuada búsqueda en la literatura; 5, Selección de estudios por duplicado; 6, Extracción de datos por duplicado; 7, Justificación de los estudios excluidos; 8, Adecuada descripción de los estudios incluidos; 9, Riesgo de sesgo de los estudios individuales incluidos; 10, Fuente de financiación de los estudios incluidos; 11, Método meta-analíticos apropiados; 12, Evaluación del riesgo de sesgo sobre el metaanálisis; 13, Consideración del riesgo de sesgo en la interpretación de los resultados de la revisión; 14, Explicación de heterogeneidad en los resultados.; 15, Evaluación de la presencia y el impacto del sesgo de publicación; 16, Conflicto de intereses; Y, *Yes*; N, *No*; NA, *Non answered*; PY, *Partial Yes*; AMSTAR-2, *A Messurement Tool to Assess Systematic Review*.

**Tabla 2.** **Resumen de las revisiones sistemáticas y metaanálisis incluidos sobre el uso de dispositivos robóticos para el entrenamiento de la marcha en el ictus**.

Trabajo	Tipo de estudio	Actualización	Población	Sujetos	Intervenciones	Comparación	Medida de resultado	Resultados	Conclusiones	Limitaciones
Cho *et al*. (2018) [15]	Revisión sistemática	Abril de 2016	Sujetos que hayan sufrido un ictus. >18 años en fase aguda/subaguda, que se encuentre dentro de los 3 primeros meses.	N = 220	Robot de asistencia a la marcha en combinación con fisioterapia:	Terapia rehabilitadora convencional.	Velocidad de la marcha (10MWT).	Resultados descriptivos	No se puede concluir que el entrenamiento de la marcha con dispositivos robóticos sea superior al entrenamiento convencional. Sin embargo, se observan beneficios cuando se combinan ambos tratamientos.	Solo incluyen estudios en un único idioma, inglés.
					*Lokomat* (n = 3).		Movilidad funcional (RMI, FAC y TUG).			Bajo número de estudios.
					*G-EO system* (n = 1).		Resistencia (6MWT).			Bajo número de participantes.
					*Walk-around gaiter* (n = 1).					Los estudios incluidos presentan niveles Riesgo de sesgo de cegamiento en participantes, terapeutas y evaluadores.
					*Gait Trainer* (n = 1).					
					*Gait-assistance robot* (GAR) (n = 1).					
Bruni *et al*. (2018) [13]	Metaanálisis	Junio de 2015	Sujetos que hayan sufrido un ictus.	N = 673	Robot de asistencia a la marcha:	Fisioterapia convencional.	Velocidad de la marcha (10–5MWT).	Velocidad: 0,38 (0,21–0,55) (DME)	El uso de un entrenamiento de la marcha mediante dispositivos robóticos afecta positivamente a los resultados de la marcha evaluados.	Bajo número de estudios.
					Exoesqueleto*: Lokomat* (n = 6)		Movilidad funcional (FAC).	*p * < 0,05		No realiza un análisis de riesgo de sesgos de los estudios incluidos (al menos no lo menciona).
					Efector final*: Gait trainer* (n = 7)			FAC: –0,80 (–1,14–(–0,46)) (DME)		
								*p * > 0,05		Bajo el tamaño muestral de los estudios incluidos.
Postol *et al*. (2019) [14]	Metaanálisis	Julio de 2017	Sujetos que hayan sufrido un ictus. >18 años, independientemente del tiempo y del nivel de discapacidad.	N = 322	Robot de asistencia a la marcha.	Terapia convencional.	Velocidad de la marcha (10MWT).	Velocidad: 0,73 m/s	No se observan mejoras significativas en el entrenamiento de la marcha mediante dispositivos robóticos frente a la terapia convencional.	Heterogeneidad en el diseño de los estudios.
					*Hybrid assistive limb* (HAL) (n = 7).		Resistencia (6MWT).	(0,03–1,43) (DM)		Bajo número de estudios.
					Tibion bionic Leg (n = 4).			*p * > 0,05		Bajo tamaño muestral.
					*Stride management Assist* (SMA) (n = 1).			Resistencia: 6,52 metros		Incluye estudios no controlados.
					*H2 skeleton* (n = 1).			(–18,01–31,04) (DM)		Incluye estudios que omiten la descripción de la terapia.
								*p * > 0,05		Heterogeneidad en dosis de terapia, tratamientos, seguimientos y estado de los pacientes incluidos.
										Calidad metodológica variable.
Tedla *et al*. (2019) [16]	Metaanálisis	2018	Sujetos que hayan sufrido ictus. >18 años, independientemente del tiempo y del nivel de discapacidad.	-	Robot de asistencia a la marcha en combinación de fisioterapia convencional (n = 4) o en solitario (n = 5):	Terapia convencional, entrenamiento de marcha asistida por terapeutas y grupo de entrenamiento de marcha en suelo.	Velocidad de la marcha (10MWT).	Velocidad: –0,12 (–0,24–0) (DME) *p* > 0,05	No se observan mejoras significativas en el entrenamiento de la marcha mediante dispositivos robóticos frente a la terapia convencional.	Bajo número de estudios.
										Bajo número de participantes.
					*Lokomat* (n = 7).					Heterogeneidad en dosis de terapia, tratamientos, seguimientos y estado de los pacientes incluidos.
					*Gait assistance robot* (n = 1).					
					*Gait trainer robot* (n = 1).					
										Heterogeneidad en el metaanálisis en la comparación de terapias combinadas y en solitario.
Maranesi *et al*. (2020) [33]	Revisión sistemática	Octubre de 2018	Pacientes que hayan sufrido un ictus igual o > de 60 años.	N = 591	Tecnología efectora final de asistencia a la marcha con o sin estimulación eléctrica funcional.	Fisioterapia convencional de entrenamiento de marcha.	Velocidad de la marcha (10MWT).	Resultados descriptivos	Un entrenamiento en combinación con terapia convencional parece mejorar la capacidad de la marcha.	Falta de datos a largo plazo.
						Exoesqueleto y efector final (n = 1).	Movilidad funcional (FAC y RMI).			Heterogeneidad en las medidas de resultado.
							Resistencia (6MWT).			Heterogeneidad en protocolos de intervención.
										Sin protocolo registrado.
										No selección ni extracción de datos por duplicado.
Schröder *et al*. (2019) [31]	Metaanálisis	24 de octubre de 2017	Sujetos mayores de 18 años que hayan sufrido un ictus.	N = 863	Repetitive gait training (RGT).	Fisioterapia convencional.	Velocidad de la marcha (10MWT).	Velocidad: 0,05	Los dispositivos robóticos permiten un entrenamiento más intensivo de la marcha que un tratamiento convencional, que podría mejorar la capacidad funcional.	Heterogeneidad en protocolos de intervención.
					RAGT Exo (*Repetitive Assistance Gait Trainer*).		Movilidad funcional (FAC).	(0–0,11) (DM)		Estudios incluidos con sesgos de cegamiento.
					RAGT EE (*Repetitive Assistance Gait Trainer*).		Resistencia 6MWT).	*p * > 0,05		En el análisis estadístico incluye exoesqueleto, efector final y cinta de marcha.
							Función motora (FMA).	Movilidad: 0,38		
					BWST.			(–0,03–0,78) (DM)		
							Fuerza muscular (MI-LE).	*p * > 0,05		
								Resistencia: 24,36 metros		
								(3,58–45,14) (DM)		
								*p * > 0,05		
								Función motora: 0,52		
								(–1,54–2,59) (DM)		
								*p * > 0,05		
								Fuerza: 3,64		
								(–2,88–10,57) (DM)		
								*p* = 0.008		
Mehrholz *et al*. (2020) [34]	Metaanálisis	6 de enero de 2020	Sujetos mayores de 18 años que hayan sufrido un ictus.	N = 2440	Robot de asistencia a la marcha:	Terapia convencional.	Velocidad de la marcha (10MWT).	Velocidad: 0,06 (0,02–0,1) (DM)	La combinación de dispositivos robóticos y terapia convencional es superior al entrenamiento robótico aislado, ya que potencia la recuperación funcional y aumenta la probabilidad de lograr una marcha independiente.	Incluye estudios no acabados.
					*G-EO system® (n = 4); Lokomat® (n = 25); Ekso® (n = 1); Stride management assist® (SMA) (n = 3); Gait trainer® (n = 9); Robo-gait® (n = 1); AutoAmbulator (n = 1); Anklebot® (n = 1); Walkbot® (n = 1); Morning walk® (n = 1); Exowalk® (n = 3); Gait Enhancing and Motivating System® (n = 1); Robot-assisted device (n = 1); Gait-assistance robot (n = 1); Bionic leg device® (AlterG) (n = 1); Gait master4® (n = 1); Gear system® (n = 1).*	Terapia Bobath.	Movilidad funcional (FAC).	*p* = 0,004**		Heterogeneidad en dosis, tipo de terapia, tratamientos, seguimientos, cegamientos y aleatorización.
						Entrenamiento dirigido a tareas funcionales.	Resistencia (6MWT).	Movilidad: 2,14 (1,57–2,92) (OR)		
								*p * < 0,0001***		Incluye estudios con riesgo de sesgos.
						Terapia de marcha asistida por terapeutas.		Resistencia: 10,86 metros (–21,47–36,99) (DM)		
								*p * > 0,05		
					*Ankle device (n = 1); Hybrid assistive limb® (HAL) (n = 3); Exoskeleton and ankle robot (n = 1).*					
Moucheboeuf *et al*. (2020) [32]	Metaanálisis	Noviembre de 2019	Pacientes adultos diagnosticados de ictus.	N = 1466	Robot de asistencia a la marcha en combinación con fisioterapia convencional, entrenamiento con suspensión parcial del peso y otras terapias.	Fisioterapia convencional.	Velocidad de la marcha (10MWT).	Velocidad: 0,09 m/s (0,03–0,15) (DM)	La combinación de robótica, terapia convencional y soporte de peso mejora la velocidad especialmente en pacientes dependientes.	Heterogeneidad en protocolos de intervención. Heterogeneidad en la selección de los participantes en los estudios. Riesgo de sesgo.
						BWST.	Movilidad funcional (FAC y TUG).	*p * < 0,05		
						RAGT.	Resistencia de la marcha (6MWT).	Movilidad (FAC): 0,51 (0,07–0,95) (DM)	Se recomienda una intensidad mínima de 20 sesiones (una hora diaria).	En análisis estadístico combina todo tipo de sistemas robóticos.
								*p * < 0,05		
								Movilidad (TUG): 3,2 s (–3,2–8,98) (DM)		
								*p * > 0,05		
								Resistencia: 23,75 metros (–16,9–62,35) (DM)		
								*p * > 0,05		
Hsu *et al*. (2020) [30]	Metaanálisis	1 de julio de 2019	Pacientes diagnosticados de ictus (6 meses).	N = 1452	Tecnología asistida de marcha:	Fisioterapia convencional.	Velocidad de la marcha (10MWT).	Velocidad: 0,01 (–0,08–0,09) (DME)	No se encuentran diferencias en el entrenamiento de la marcha mediante dispositivos robóticos y terapia convencional.	Heterogeneidad en protocolos de intervención.
					RAGT.		Movilidad funcional (FAC, TUG, RMI y mEFAP).	*p * > 0,05		Heterogeneidad en dispositivos de marcha.
					BWST.			Movilidad: 0,19 (–0,1–0,49) (DME) *p* > 0,05		
							Resistencia de la marcha (6MWT).			
								Resistencia: –0,4 (–0,36–0,28) (DME)		
								*p * > 0,05		
Nedergård *et al*. (2021) [28]	Metaanálisis	19 de enero de 2019	Pacientes con ictus >18 años.	N = 412	Entrenamiento mediante robot asistido de marcha.	Otras intervenciones de fisioterapia.	Velocidad de la marcha.	Velocidad: 0 (–0,05–0,05) (DM)	Existe falta de evidencia en la mejora de la marcha en el entrenamiento mediante dispositivos robóticos.	Bajo tamaño muestral.
							Cadencia.	*p * > 0,05		Heterogeneidad en protocolos de intervención y en medidas de resultado.
							Longitud de paso.	Cadencia: 1,44 (–2,34–5,22) (DM)		
							Longitud de zancada.	*p * < 0,05		Solo estudios en inglés.
								Longitud paso:1,22 (–0,1–2,54) (DM)		
								*p * > 0,05		
								Longitud zancada: 2,86 (0,46–5,25) (DM)		
								*p * < 0,05		
Calafiore *et al*. (2022) [26]	Metaanálisis	18 de enero de 2021	Pacientes de ictus en fase subaguda (<6 meses).	N = 576	Todo tipo de dispositivos exoesqueleto robóticos	Fisioterapia convencional.	Movilidad funcional (FAC).	Movilidad: –0,09 (–0,22–0,03) (DM)	No se demuestra que el entrenamiento de la marcha mediante dispositivos robóticos sea superior al uso de la terapia convencional.	Bajo tamaño muestral.
								*p * > 0,05		Heterogeneidad en protocolo de intervención.
Hsu *et al*. (2023) [27]	Metaanálisis	31 de julio de 2021	Pacientes con ictus.	N = 492	Exoesqueleto portátil.	Fisioterapia convencional.	Velocidad de la marcha (10MWT).	Velocidad: 0,13 (0,05–0,21) (DME)	El entrenamiento de la marcha mediante dispositivos robóticos demostró superioridad al uso de la terapia convencional.	Bajo tamaño muestral.
							Movilidad funcional (FAC).	*p * < 0,05		Heterogeneidad en protocolos de intervención.
							Resistencia (6MWT).	Movilidad: 0,16 (–0,15–0,48) (DME) *p* > 0,05		Solo estudios en inglés.
							Función motora (FMA).			
								Resistencia: 0,15 (–0,09–0,39) (DME)		
								*p * > 0,05		
								Función motora: 20,35 (–4,08–44,77) (DME)		
								*p * > 0,05		
Zhang *et al*. (2023) [23]	Metaanálisis	31 de agosto de 2022	Pacientes con ictus >18 años.	N = 1571	Dispositivo robótico de asistencia a la marcha y RV, combinada o en solitario.	Fisioterapia convencional.	Velocidad de la marcha.	Velocidad: 0,88 (–0,86–2,66) (DM) *p* > 0,05	No se observan mejoras significativas de la marcha en el entrenamiento mediante dispositivos robóticos.	Bajo tamaño muestral.
							Cadencia.			Heterogeneidad en protocolos de intervención.
								Cadencia: 3,94 (–1,25–11,55) (DM)		
								*p * > 0,05		

RAGT, repetitive assistance gait trainer; BWST, body weight support training; FAC, Functional Ambulatory Category; TUG, Time Up and Go; FMA, Fugl Meyer Assessment; mEFAP, modified Emory Functional Ambulatory Profile; 6MWT, 6 Minutes walking test; 10MWT, 10 meters walking test; MAS, Modified Ashworth Scale; RMI, Rivermead Mobility Index; MI-LE, Motricity Index Lower-Extremity; DM, diferencia de medias; DME, diferencia de medias estandarizadas; OR, odd ratio. ** *p* ≤ 0,01; *** *p* ≤ 0,001.

Las intervenciones se aplicaron como tratamiento único o combinadas con otros tratamientos de fisioterapia (entrenamiento sobre cinta de marcha y fisioterapia convencional). Los parámetros de intervención variaron entre los estudios:

- Duración: de 10 días a 16 semanas.
- Frecuencia: de 2 a 5 días/semana.
- Rango de sesiones: de 10 a 96 sesiones.
- Duración de las sesiones: de 20 a 105 minutos.

Las medidas de resultados descritas por las revisiones se organizaron según los siguientes desenlaces: Parámetros espaciotemporales de la marcha [13, 14, 15, 16, 23, 26, 27, 28, 30, 31, 32, 33, 34]. Movilidad funcional [13, 15, 23, 26, 27, 30, 31, 32, 33, 34]. Resistencia de la marcha [14, 15, 27, 30, 31, 32, 33]. Función motora [27, 31], y fuerza muscular [31].

### 3.1 Parámetros Espaciotemporales

#### 3.1.1 Exoesqueletos no Portátiles

Las medidas de resultado descritas fueron el 10 meter walk test (10MWT) y el análisis cuantitativo mediante sistemas tecnológicos. En cuanto a la velocidad de la marcha, las revisiones de Bruni *et al*. [13], Tedla *et al*. [16], Cho *et al*. [15] y Mehrholz *et al*. [34], no encontraron diferencias significativas del entrenamiento de la marcha con exoesqueletos no portables en comparación con el tratamiento convencional. No obstante, el estudio de Zhang *et al*. [23] sí reportó una mejora significativa en la velocidad de la marcha evaluada mediante sistemas tecnológicos, en contraste con fisioterapia convencional. La certeza de la evidencia se clasificó como baja y moderada.

#### 3.1.2 Efectores Finales

El metaanálisis de Bruni *et al*. [13] y Mehrholz *et al*. [34] mostraron una mejora significativa en la velocidad de la marcha a favor del uso de dispositivos de efector final, aunque con una baja certeza de la evidencia. En contraste, la revisión de Maranesi *et al*. [33], que presentó una alta imprecisión debido a la inclusión de un único estudio, no encontró un beneficio significativo en comparación con el tratamiento convencional.

#### 3.1.3 Exoesqueleto Portátil

El estudio de Postol *et al*. [14] describió beneficios para el grupo control; sin embargo, el estudio de Hsu *et al*. [27], a pesar de contar con una baja certeza en la evidencia, encontró diferencias significativas en la velocidad de la marcha a favor del uso de estos dispositivos en comparación con la terapia convencional.

#### 3.1.4 Dispositivos Robóticos Combinados

Por último, los estudios incluidos que combinaron distintos tipos de dispositivos robóticos, Hsu *et al*. [30], Nedergård *et al*. [28] y Schröder *et al* [31], no encontraron diferencias significativas en la velocidad de la marcha respecto al grupo control. Sin embargo, los estudios de Moucheboeuf *et al*. [32] y Mehrholz *et al*. [34] reportaron mejoras significativas con un alto nivel de evidencia.

Los parámetros de cadencia, longitud de paso y de zancada fueron analizados por el estudio de Nedergård *et al*. [28], que solo encontró diferencias significativas en la longitud de zancada, aunque con una certeza de la evidencia muy baja.

### 3.2 Movilidad Funcional

#### 3.2.1 Exoesqueleto no Portable

La medida de resultado evaluada fue la FAC (*Functional Ambulation Category*)*.* De los cuatro estudios que evaluaron la capacidad de deambulación independiente, Schröder *et al*. [31], Cho *et al*. [15] y Calafiore *et al*. [26] no reportaron diferencias significativas. En contraste, Mehrholz *et al*. [34] con una certeza de la evidencia moderada, sí encontró diferencias significativas.

#### 3.2.2 Efectores Finales 

Las medidas de resultado fueron la FAC y la RMI (*Rivermead Mobility Index*). De las cuatro revisiones que realizaron un análisis para evaluar la capacidad de marcha independiente, Bruni *et al*. [13], Maranesi *et al*. [33] y Schröder *et al*. [31], reportaron diferencias significativas con una certeza de la evidencia moderada y baja. Por otro lado, aunque los resultados de Mehrholz *et al*. [34] favorecieron al grupo de intervención, las diferencias no fueron estadísticamente significativas. La certeza de la evidencia para esta variable fue clasificada como moderada.

#### 3.2.3 Exoesqueleto Portátil

Las medidas de resultado fueron la FAC y el *Timed Up and Go* (TUG). El estudio de Hsu *et al*. [27], con una certeza de la evidencia baja, no reportaron diferencias significativas frente a la terapia convencional.

#### 3.2.4 Dispositivos Robóticos Combinados

Se evaluaron la FAC, el TUG, el *modified Emory Functional Ambulatory Profile* (mEFAP) y la RMI en relación con el entrenamiento de marcha mediante dispositivos robóticos. Dos metaanálisis (Moucheboeuf *et al*. [32] y Hsu *et al*. [30]) con baja certeza de evidencia no mostraron diferencias significativas entre los grupos. Sin embargo, Mehrholz *et al*. [34], con alta certeza de la evidencia, sí encontraron diferencias significativas a favor del uso combinado de dispositivos robóticos frente a la terapia convencional.

### 3.3 Resistencia de Marcha

#### 3.3.1 Exoesqueleto

La medida de resultado evaluada fue el *6-Minutes Walk Test* (6MWT). En el estudio de Mehrholz *et al*. [34], con una certeza de la evidencia moderada, los resultados favorecieron al grupo control, aunque no se demostraron diferencias significativas. Por su parte, la revisión de Cho *et al*. [15], que incluyó un único estudio para este resultado, no pudo determinar un beneficio claro para ninguno de los grupos.

#### 3.3.2 Efectores Finales

Dos metaanálisis, Schröder *et al*. [31] y Mehrholz *et al*. [34] con una certeza de la evidencia muy baja y moderada, respectivamente, mostraron beneficios significativos a favor del grupo de intervención. Sin embargo, la revisión de Maranesi *et al*. [33] no encontraron diferencias significativas a favor del uso de un efector final.

#### 3.3.3 Exoesqueleto Portátil

Mientras que, el estudio de Postol *et al*. [14] reportó un beneficio para el uso de terapia convencional, el estudio de Hsu *et al*. [27], con una certeza de la evidencia muy baja, reportó beneficios no significativos frente a la terapia convencional.

#### 3.3.4 Dispositivos Robóticos Combinados

Aunque todos los estudios incluidos-Moucheboeuf *et al*. [32], Hsu *et al*. [30] y Mehrholz *et al*. [34]-describen un beneficio para el grupo de intervención, ninguno reportó diferencias significativas en comparación con el grupo control.

### 3.4 Función Motora y Fuerza

#### 3.4.1 Exoesqueleto y Exoesqueletos Portátiles 

La medida de resultado descrita fue la *Fugl-Meyer Assessment* (FMA). El estudio de Schröder *et al*. [31] que evaluó el uso de un exoesqueleto no portables para la mejora de la función motora, no reportaron diferencias significativas a favor de este dispositivo. De manera similar, el estudio de Hsu *et al*. [27], que analizó el uso de un exoesqueleto portable, tampoco mostraron mejoras significativas para el grupo de intervención. Ambos estudios presentaron una certeza de la evidencia muy baja.

#### 3.4.2 Efectores Finales

La medida de resultado descrita fue el *Motricity Index Lower-Extremity* (MI-LE). Solo un estudio, Schröder *et al*. [31], evaluó la fuerza muscular de los pacientes tras el uso de un dispositivo de efector final con una certeza de la evidencia muy baja, el estudio reportó una mejora significativa a favor del grupo de intervención.

### 3.5 Decisión Sobre Utilizar un Dispositivo Robótico para la Mejora de la Marcha en Pacientes con Ictus

De acuerdo con el procedimiento de evaluación GRADE, la recomendación clínica ofrecida sobre la intervención a estudio en la presente revisión de revisiones sistemáticas debe fundamentarse en los trabajos que han obtenido la mejor evaluación de su calidad metodológica.

Primero, se seleccionó el metaanálisis de Mehrholz *et al*. [34] para las medidas de resultado de velocidad de la marcha, movilidad funcional y resistencia, por ser el más representativo. Segundo, para la función motora y la fuerza muscular, se utilizó el metaanálisis de Schröder *et al*. [31]. Por último, de forma específica para los dispositivos portátiles, el estudio de Hsu *et al*. [27] (véanse Tabla 3, Ref. [35] y Tabla 4, Ref. [27, 31, 34]).

**Tabla 3.** **Decisión de recomendación clínica para el entrenamiento mediante dispositivos robóticos**.

PREGUNTA	
¿Debería usarse un dispositivo robótico de asistencia a la marcha en combinación con fisioterapia versus fisioterapia convencional para mejorar la marcha en pacientes de ictus?	
POBLACIÓN	Pacientes con ictus.
INTERVENCIÓN	Un dispositivo robótico de asistencia a la marcha en combinación con fisioterapia.
COMPARACIÓN	Fisioterapia convencional.
DESENLACES	Velocidad de la marcha, movilidad funcional, resistencia, función motora y fuerza.
TIPO DE RECOMENDACIÓN	Recomendación condicional a favor de la intervención.
RECOMENDACIÓN	El panel sugiere que realizar un tratamiento mediante entrenamiento de la marcha usando dispositivos robóticos en combinación con otra técnica podría mejorar algunos parámetros de la marcha como la velocidad y la capacidad de caminar independientemente, sin embargo, otras técnicas más económicas y accesibles para el profesional y el paciente pudieran resultar en los mismos resultados. Aunque la certeza de la evidencia varíe en función del desenlace, y los costes sean variables, el análisis estadístico refiere un beneficio para el uso de estas técnicas. Por tanto, el panel se inclina por emitir una recomendación condicional a favor de estas técnicas.
JUSTIFICACIÓN GLOBAL	Debido a los resultados estadísticos, la certeza de la evidencia, los beneficios de estos tratamientos, especialmente a la hora de potenciar y ayudar a la intensidad en la terapia, el panel realizó una recomendación condicional a favor del uso de un entrenamiento de marcha mediante dispositivos robóticos.
JUSTIFICACIÓN DETALLADA	*Certeza de la evidencia:*
	Aunque la certeza de la evidencia global varía a lo largo de las medidas de resultados, uno de los resultados críticos, como la movilidad funcional, presentó una certeza de la evidencia alta, lo que apoya su recomendación.
	*Balance de efectos:*
	Dado que los estudios incluidos no evidenciaron efectos adversos relevantes, y los beneficios favorecieron en desenlaces críticos como la independencia funcional, el balance de efectos favorece la intervención.
	*Recursos:*
	El coste asociado a la terapia robótica varía en función del tipo de dispositivo. La terapia con efector final (490 €) es similar en coste a la convencional (480 €), mientras que la terapia con exoesqueleto representa un coste significativamente mayor (1353 € por paciente). No se dispone de estimaciones para dispositivos portables. Estos costes deben valorarse en función del beneficio clínico obtenido [35].
	*Coste-eficacia:*
	La evidencia sugiere que el uso de un efector final para el entrenamiento de la marcha presenta un balance coste-efectivo mayor que el uso de una terapia convencional, especialmente en la recuperación de la independencia y la velocidad de la marcha. Sin embargo, el uso de un exoesqueleto, aunque menos eficiente en términos económicos, presenta una ventaja adicional, ya que puede ser implementada en un mayor rango de pacientes, incluyendo aquellos con peor pronóstico funcional [35].
	*Equidad:*
	Actualmente, existen posibles desigualdades en la aplicación de estos dispositivos que se asocian a su alto coste y su implementación limitada a ciertos entornos, generalmente a clínicas privadas. Además, el beneficio clínico puede variar en función del pronóstico del paciente, lo que podría generar inequidades. Por lo tanto, esto reduce una recomendación fuerte a favor de estos dispositivos.
	*Aceptabilidad:*
	Algunos terapeutas podrían ser reacios al uso de estos dispositivos robóticos debido al tiempo, la especialización que requiere y la variabilidad en los beneficios. Además, algunos profesionales podrían tomar la decisión de aplicar otros tratamientos más sencillos en pacientes con mal pronóstico.

*Exchange rate reference (March 13, 2026): 1 EUR = 1.09 USD/0.85 GBP (Source: European Central Bank)*.

**Tabla 4.** **Resumen de la evidencia para el entrenamiento de la marcha mediante dispositivos robóticos para pacientes con ictus**.

Estudio	N	Intervención	Grupo control	Medida de resultado	DM	IC	*p* valor	Calidad de la evidencia	Certeza de la evidencia	Decisión clínica	Recomendación clínica global
Mehrholz *et al*. [34]	742	Exoesqueleto	Tratamiento convencional	Velocidad de la marcha	0 (DM)	–0,05–0,04	0,870	Alta	⊕⊕⊕○ Moderada	Recomendación condicional para la intervención o la comparación	Recomendación condicional para la intervención
Mehrholz *et al*. [34]	665	Efector final	Tratamiento convencional	Velocidad de la marcha	0,12 (DM)	0,05–0,19	0,001***	Alta	⊕⊕○○ Baja	Recomendación condicional para la intervención	
Hsu *et al*. [27]	385	Exoesqueleto portátil	Tratamiento convencional	Velocidad de la marcha	0,13 (DM)	0,05–0,21	0,002**	Baja	⊕⊕○○ Baja	Recomendación condicional para la intervención	
Mehrholz *et al*. [34]	1600	Dispositivo robótico combinado	Tratamiento convencional	Velocidad de la marcha	0,06 (DM)	0,02–0,1	0,004**	Alta	⊕⊕○○ Baja	Recomendación condicional para la intervención	
Mehrholz *et al*. [34]	685	Exoesqueleto	Tratamiento convencional	FAC	2,11 (OR)	1,36–3,29	0,001***	Alta	⊕⊕⊕○ Moderada	Recomendación condicional para la intervención	
Hsu *et al*. [27]	395	Exoesqueleto portátil	Tratamiento convencional	TUG y FAC	0,16 (DME)	–0,15–0,48	0,320	Baja	⊕⊕○○ Baja	Recomendación condicional para la intervención o la comparación	
Mehrholz *et al*. [34]	598	Efector final	Tratamiento convencional	FAC	1,90 (OR)	0,99–3,63	0,050	Alta	⊕⊕⊕○ Moderada	Recomendación condicional para la intervención o la comparación	
Mehrholz *et al*. [34]	1567	Dispositivo robótico combinado	Tratamiento convencional	FAC	2,14 (OR)	1,57–2,92	<0,001***	Alta	⊕⊕⊕⊕ Alta	Recomendación condicional para la intervención	
Hsu *et al*. [27]	367	Exoesqueleto portátil	Tratamiento convencional	6MWT	–0,04 (DME)	–0,36–0,28	0,820	Baja	⊕⊕○○ Baja	Recomendación condicional para la intervención o la comparación	
Mehrholz *et al*. [34]	468	Exoesqueleto	Tratamiento convencional	6MWT	–8,32 (DM)	–27,32–11,08	0,400	Alta	⊕⊕⊕○ Moderada	Recomendación condicional para la intervención o la comparación	
Mehrholz *et al*. [34]	416	Efector final	Tratamiento convencional	6MWT	31,22 (DM)	10,35–52,08	0,003**	Alta	⊕⊕⊕○ Moderada	Recomendación condicional para la intervención	
Hsu *et al*. [27]	278	Exoesqueleto portátil	Tratamiento convencional	FMA	0,15 (DME)	–0,09–0,39	0,220	Baja	⊕○○○ Muy baja	Recomendación condicional para la intervención o la comparación	
Schröder *et al*. [31]	119	Exoesqueleto	Tratamiento convencional	FMA	0,76 (DM)	–1,83–3,36	0,560	Baja	⊕○○○ Muy baja	Recomendación condicional para la intervención o la comparación	
Schröder *et al*. [31]	230	Efector final	Tratamiento convencional	MI-LE	8 (DM)	2,08–13,93	0,008**	Baja	⊕○○○ Muy baja	Recomendación condicional para la intervención	

IC, Intervalo de Confianza; Significación estadística; ** *p* ≤ 0,01; *** *p* ≤ 0,001; ⊕○○○, muy baja ⊕⊕○○, baja; ⊕⊕⊕○, moderada; ⊕⊕⊕⊕, alta.

## 4. Discusión

El presente trabajo constituye una revisión de revisiones sistemáticas y metaanálisis cuyo objetivo es analizar la calidad de la evidencia científica sobre el entrenamiento de la marcha basado en dispositivos robóticos en pacientes con ictus.

El entrenamiento de la marcha con dispositivos robóticos permite un trabajo intensivo y repetitivo, lo que a priori, podría favorecer la activación de las regiones motoras cerebrales relacionadas con el movimiento y estimular así, los procesos neuroplásticos [36]. Sin embargo, su efectividad sigue siendo un tema de debate debido a la disparidad de los resultados de los estudios científicos y a la falta de evidencia sólida sobre su impacto a largo plazo. La cantidad de literatura científica sobre esta cuestión también es extensa y dificulta responder a esta cuestión. Por ello, se considera necesario agruparla, analizarla y clasificarla.

Es importante profundizar en las diferencias claves en el uso de los distintos dispositivos robóticos para la recuperación de la marcha del paciente con ictus. Los dispositivos robóticos de efector final reproducen el patrón de marcha a nivel distal sin alinear las articulaciones del miembro inferior [33]. Sin embargo, al no brindar soporte a las articulaciones de la rodilla y de la cadera, requieren mayor control motor del paciente, siendo limitada su aplicación en pacientes con menor funcionalidad [37]. Los exoesqueletos portátiles permiten caminar en entornos reales, lo que favorece una mejor integración de la marcha en las actividades de la vida diaria. No obstante, su eficacia puede verse limitada en pacientes con déficits cognitivos o motores severos, ya que estos dispositivos requieren un mayor control del equilibrio y una atención sostenida por parte del usuario [27].

Según los resultados de la presente revisión de revisiones sistemáticas y metaanálisis, los dispositivos robóticos de efector final generan mejoras en la velocidad de la marcha; sin embargo, los exoesqueletos portables y no portables presentan beneficios discretos o no significativos [13, 14, 16, 27, 34]. Si bien, estos cambios podrían considerarse relevantes desde una perspectiva clínica, solo los efectores finales se acercan al umbral del cambio clínicamente significativo establecido para la velocidad en pacientes con ictus (superior a 0.16 m/s) [38, 39]. De forma específica, velocidades entre 0.4–0.8 m/s se asocian con una mayor participación comunitaria, por tanto, la mejora en la velocidad de marcha obtenida con los dispositivos robóticos en los trabajos incluidos en la presente revisión (entre 0.06 y 0.12 m/s) puede no ser suficiente para que los pacientes experimenten un cambio funcional. Esto sugiere que, aunque el entrenamiento con dispositivos robóticos puede contribuir a la recuperación de la marcha, su impacto en la reinserción comunitaria podría ser limitado en aquellos sujetos que presentan una velocidad inicial reducida [38].

En cuanto a la recuperación de la marcha independiente, estos dispositivos parecen tener un impacto positivo en pacientes en fases aguda y subaguda con alta dependencia inicial, pero con menos repercusión en la población crónica, lo que plantea dudas sobre su aplicabilidad en esta fase del ictus [27, 34]. Estas mejoras en la independencia en las fases agudas y subagudas podrían reducir el riesgo de caídas asociado a esta población [40]. En este contexto, la recuperación de la marcha se evaluó con frecuencia mediante la escala FAC y se analizó como variable dicotómica (*odds ratio*), observándose mayores probabilidades de alcanzar la marcha independiente en pacientes en fase aguda/subaguda y con mayor dependencia inicial, mientras que en la fase crónica el beneficio no resulta tan claro.

En términos de resistencia, según las revisiones incluidas, los pacientes con ictus que entrenaron con efectores finales pueden recorrer hasta 31 metros más que el grupo control; mientras que, los pacientes que usan exoesqueletos y exoesqueletos portátiles solo alcanzan mejoras que oscilan entre 8 y 20 metros, respectivamente, sin diferencias relevantes [27, 31, 34]. Dado que el cambio mínimamente detectable para la resistencia de marcha en pacientes con ictus oscila entre 36–60 metros y el cambio clínicamente significativo entre 34–50 metros, estos resultados sugieren que, el entrenamiento de la marcha con dispositivos robóticos no logra un impacto funcional relevante en términos de distancias recorridas [39, 41, 42].

Respecto a los efectos en la función motora, los trabajos incluidos ofrecen una recomendación limitada y no muestran que, los dispositivos robóticos tengan ventajas superiores al entrenamiento convencional de la marcha [27, 31].

En cuanto a los protocolos de intervención, no hay suficiente evidencia científica para recomendar un enfoque óptimo, aunque se sugiere un mínimo de 18 sesiones de 30-45 minutos, al menos tres veces por semana [9, 43].

Por último, en relación con la calidad metodológica, únicamente el trabajo de Mehrholz *et al*. [34] fue clasificado como de alta calidad, mientras que el resto de las revisiones sistemáticas presentaron limitaciones críticas según los criterios de AMSTAR-2 [21]. Entre estas deficiencias destacan la ausencia de un protocolo previo, la insuficiencia en la búsqueda bibliográfica y la evaluación inadecuada del riesgo de sesgo o de los sesgos de publicación. Estas deficiencias pueden llevar a un reporte selectivo de resultados o análisis post hoc que sesguen las conclusiones. Además, los ECA incluidos en las revisiones sistemáticas mostraron heterogeneidad en cuanto al diseño, las intervenciones y las características de los participantes, lo que dificulta la comparación de los resultados. Por último, la variabilidad en las medidas de resultado y en los métodos de aleatorización y cegamiento refuerza la necesidad de un enfoque más homogéneo. Si bien, en la mayoría de los casos los dispositivos robóticos coexistieron durante el tratamiento con la terapia convencional, su alto coste, las limitaciones metodológicas de los trabajos científicos evaluados y la falta de estudios de costo-efectividad plantean dudas sobre su viabilidad clínica.

Este trabajo presenta una serie de limitaciones que deben ser consideradas. En primer lugar, solo se incluyeron estudios publicados en inglés y/o español, no se llevó a cabo una búsqueda exhaustiva de la literatura gris. Asimismo, los resultados podrían no ser extrapolables a poblaciones con perfiles clínicos complejos, ya que no se consideraron condiciones específicas asociadas a complicaciones en la recuperación, como cardiopatías preexistentes o pacientes de edad avanzada (mayores de 85 años) [44, 45, 46]. Finalmente, aunque se ha intentado seguir un enfoque riguroso y sistemático, no puede descartarse la posibilidad de errores de selección, extracción o interpretación de los datos.

Futuros estudios deberían considerar comparar la eficacia de la robótica según la localización y el subtipo etiológico de ictus, como los infartos lacunares capsulares o pontinos. Asimismo, se recomienda la inclusión de poblaciones específicas con mayor limitación funcional, evaluar el coste-efectividad y determinar la dosis terapéutica óptima según el estadio clínico.

## 5. Conclusiones

El entrenamiento de la marcha asistido por dispositivos robóticos en pacientes con ictus muestra ciertos beneficios potenciales, especialmente en términos de velocidad de la marcha, cuando se combina con otras intervenciones terapéuticas. Sin embargo, la evidencia disponible no permite establecer conclusiones firmes sobre su efectividad clínica general, debido a la heterogeneidad de los estudios, la baja calidad metodológica de muchas revisiones sistemáticas y la ausencia de mejoras funcionales consistentes en parámetros clave como la resistencia de marcha o la función motora.

Los dispositivos de efector final parecen ser los que ofrecen resultados más prometedores, aunque estos siguen estando por debajo de los umbrales de cambio clínicamente significativo en muchas variables. Los exoesqueletos portables y no portables muestran beneficios más modestos y su eficacia puede verse limitada por las demandas cognitivas y motoras que implican.

La aplicabilidad de estos dispositivos también se ve restringida por su alto coste, su limitada accesibilidad y la falta de estudios sólidos de costo-efectividad. Todo ello cuestiona su viabilidad como herramienta de uso generalizado en la práctica clínica, especialmente en fases crónicas del ictus.

## Data Availability

Están disponibles para los lectores interesados a solicitud del autor correspondiente.

## Contribuciones de los Autores

JACH: Conceptualización, Curación de datos, Investigación, Metodología, Recursos, Validación, Visualización, Redacción – borrador original, Redacción – revisión y edición, Supervisión. FMR: Conceptualización, Administración del proyecto– revisión y edición, Supervisión. MCT: Conceptualización, Administración del proyecto– revisión y edición, Supervisión. Todos los autores leyeron y aprobaron el manuscrito final. Participaron adecuadamente en el trabajo y aceptaron ser responsables de todos sus aspectos.

## Aprobación Ética y Consentimiento Informado

No aplicable.

## Financiación

Esta investigación no recibió financiación externa.

## Material Suplementario

El material suplementario asociado con este artículo se puede encontrar, en la versión en línea, en https://doi.org/10.31083/RN46880.

## References

[1] Wonsetler EC, Bowden MG (2017). A systematic review of mechanisms of gait speed change post-stroke. Part 1: spatiotemporal parameters and asymmetry ratios. *Topics in Stroke Rehabilitation*.

[2] Nagano H, Said CM, James L, Sparrow WA, Begg R (2022). Biomechanical Correlates of Falls Risk in Gait Impaired Stroke Survivors. *Frontiers in Physiology*.

[3] Alingh JF, Groen BE, Van Asseldonk EHF, Geurts ACH, Weerdesteyn V (2020). Effectiveness of rehabilitation interventions to improve paretic propulsion in individuals with stroke - A systematic review. *Clinical Biomechanics (Bristol, Avon)*.

[4] Li Y, Fan J, Yang J, He C, Li S (2018). Effects of transcranial direct current stimulation on walking ability after stroke: A systematic review and meta-analysis. *Restorative Neurology and Neuroscience*.

[5] Stinear CM, Lang CE, Zeiler S, Byblow WD (2020). Advances and challenges in stroke rehabilitation. *The Lancet. Neurology*.

[6] Eng JJ, Pastva AM (2022). Advances in Remote Monitoring for Stroke Recovery. *Stroke*.

[7] Winstein CJ, Stein J, Arena R, Bates B, Cherney LR, Cramer SC (2016). Guidelines for Adult Stroke Rehabilitation and Recovery: A Guideline for Healthcare Professionals From the American Heart Association/American Stroke Association. *Stroke*.

[8] Pournajaf S, Calabrò RS, Naro A, Goffredo M, Aprile I, Tamburella F (2023). Robotic versus Conventional Overground Gait Training in Subacute Stroke Survivors: A Multicenter Controlled Clinical Trial. *Journal of Clinical Medicine*.

[9] Koldaş Doğan Ş (2024). Robot-assisted gait training in stroke. *Turkish Journal of Physical Medicine and Rehabilitation*.

[10] Warutkar V, Dadgal R, Mangulkar UR (2022). Use of Robotics in Gait Rehabilitation Following Stroke: A Review. *Cureus*.

[11] French B, Thomas LH, Coupe J, McMahon NE, Connell L, Harrison J (2016). Repetitive task training for improving functional ability after stroke. *The Cochrane Database of Systematic Reviews*.

[12] Luo L, Meng H, Wang Z, Zhu S, Yuan S, Wang Y (2020). Effect of high-intensity exercise on cardiorespiratory fitness in stroke survivors: A systematic review and meta-analysis. *Annals of Physical and Rehabilitation Medicine*.

[13] Bruni MF, Melegari C, De Cola MC, Bramanti A, Bramanti P, Calabrò RS (2018). What does best evidence tell us about robotic gait rehabilitation in stroke patients: A systematic review and meta-analysis. *Journal of Clinical Neuroscience: Official Journal of the Neurosurgical Society of Australasia*.

[14] Postol N, Marquez J, Spartalis S, Bivard A, Spratt NJ (2019). Do powered over-ground lower limb robotic exoskeletons affect outcomes in the rehabilitation of people with acquired brain injury? Disability and Rehabilitation. *Assistive Technology*.

[15] Cho JE, Yoo JS, Kim KE, Cho ST, Jang WS, Cho KH (2018). Systematic Review of Appropriate Robotic Intervention for Gait Function in Subacute Stroke Patients. *BioMed Research International*.

[16] Tedla JS, Dixit S, Gular K, Abohashrh M (2019). Robotic-Assisted Gait Training Effect on Function and Gait Speed in Subacute and Chronic Stroke Population: A Systematic Review and Meta-Analysis of Randomized Controlled Trials. *European Neurology*.

[17] Higgins J (2012). Cochrane handbook for systematic reviews of interventions, version 5.1.0. https://handbook-5-1.cochrane.org.

[18] Sanabria AJ, Rigau D, Rotaeche R, Selva A, Marzo-Castillejo M, Alonso-Coello P (2015). GRADE: Methodology for formulating and grading recommendations in clinical practice. *Atencion Primaria*.

[19] Page MJ, McKenzie JE, Bossuyt PM, Boutron I, Hoffmann TC, Mulrow CD (2021). The PRISMA 2020 statement: an updated guideline for reporting systematic reviews. *BMJ (Clinical Research Ed.)*.

[20] Pieper D, Antoine SL, Mathes T, Neugebauer EAM, Eikermann M (2014). Systematic review finds overlapping reviews were not mentioned in every other overview. *Journal of Clinical Epidemiology*.

[21] Shea BJ, Reeves BC, Wells G, Thuku M, Hamel C, Moran J (2017). AMSTAR 2: a critical appraisal tool for systematic reviews that include randomised or non-randomised studies of healthcare interventions, or both. *BMJ (Clinical Research Ed.)*.

[22] Mc Master University (2023). GRADEpro guideline development tool software Internet. Mc Master University. https://gradepro.org.

[23] Zhang B, Wong KP, Kang R, Fu S, Qin J, Xiao Q (2023). Efficacy of Robot-Assisted and Virtual Reality Interventions on Balance, Gait, and Daily Function in Patients With Stroke: A Systematic Review and Network Meta-analysis. *Archives of Physical Medicine and Rehabilitation*.

[24] Taki S, Iwamoto Y, Imura T, Mitsutake T, Tanaka R (2022). Effects of gait training with the Hybrid Assistive Limb on gait ability in stroke patients: A systematic review of randomized controlled trials. *Journal of Clinical Neuroscience: Official Journal of the Neurosurgical Society of Australasia*.

[25] Shakti D, Mathew L, Kumar N, Kataria C (2018). Effectiveness of robo-assisted lower limb rehabilitation for spastic patients: A systematic review. *Biosensors & Bioelectronics*.

[26] Calafiore D, Negrini F, Tottoli N, Ferraro F, Ozyemisci-Taskiran O, de Sire A (2022). Efficacy of robotic exoskeleton for gait rehabilitation in patients with subacute stroke: a systematic review. *European Journal of Physical and Rehabilitation Medicine*.

[27] Hsu TH, Tsai CL, Chi JY, Hsu CY, Lin YN (2023). Effect of wearable exoskeleton on post-stroke gait: A systematic review and meta-analysis. *Annals of Physical and Rehabilitation Medicine*.

[28] Nedergård H, Arumugam A, Sandlund M, Bråndal A, Häger CK (2021). Effect of robotic-assisted gait training on objective biomechanical measures of gait in persons post-stroke: a systematic review and meta-analysis. *Journal of Neuroengineering and Rehabilitation*.

[29] Rodríguez-Fernández A, Lobo-Prat J, Font-Llagunes JM (2021). Systematic review on wearable lower-limb exoskeletons for gait training in neuromuscular impairments. *Journal of Neuroengineering and Rehabilitation*.

[30] Hsu CY, Cheng YH, Lai CH, Lin YN (2020). Clinical non-superiority of technology-assisted gait training with body weight support in patients with subacute stroke: A meta-analysis. *Annals of Physical and Rehabilitation Medicine*.

[31] Schröder J, Truijen S, Van Criekinge T, Saeys W (2019). Feasibility and effectiveness of repetitive gait training early after stroke: A systematic review and meta-analysis. *Journal of Rehabilitation Medicine*.

[32] Moucheboeuf G, Griffier R, Gasq D, Glize B, Bouyer L, Dehail P (2020). Effects of robotic gait training after stroke: A meta-analysis. *Annals of Physical and Rehabilitation Medicine*.

[33] Maranesi E, Riccardi GR, Di Donna V, Di Rosa M, Fabbietti P, Luzi R (2020). Effectiveness of Intervention Based on End-effector Gait Trainer in Older Patients With Stroke: A Systematic Review. *Journal of the American Medical Directors Association*.

[34] Mehrholz J, Thomas S, Kugler J, Pohl M, Elsner B (2020). Electromechanical-assisted training for walking after stroke. *The Cochrane Database of Systematic Reviews*.

[35] Carpino G, Pezzola A, Urbano M, Guglielmelli E (2018). Assessing Effectiveness and Costs in Robot-Mediated Lower Limbs Rehabilitation: A Meta-Analysis and State of the Art. *Journal of Healthcare Engineering*.

[36] Kim H, Park G, Shin JH, You JH (2020). Neuroplastic effects of end-effector robotic gait training for hemiparetic stroke: a randomised controlled trial. *Scientific Reports*.

[37] Hesse S, Waldner A, Tomelleri C (2010). Innovative gait robot for the repetitive practice of floor walking and stair climbing up and down in stroke patients. *Journal of Neuroengineering and Rehabilitation*.

[38] Tilson JK, Sullivan KJ, Cen SY, Rose DK, Koradia CH, Azen SP (2010). Meaningful gait speed improvement during the first 60 days poststroke: minimal clinically important difference. *Physical Therapy*.

[39] Perera S, Mody SH, Woodman RC, Studenski SA (2006). Meaningful change and responsiveness in common physical performance measures in older adults. *Journal of the American Geriatrics Society*.

[40] Alghadir AH, Al-Eisa ES, Anwer S, Sarkar B (2018). Reliability, validity, and responsiveness of three scales for measuring balance in patients with chronic stroke. *BMC Neurology*.

[41] Flansbjer UB, Holmbäck AM, Downham D, Patten C, Lexell J (2005). Reliability of gait performance tests in men and women with hemiparesis after stroke. *Journal of Rehabilitation Medicine*.

[42] Fulk GD, He Y (2018). Minimal Clinically Important Difference of the 6-Minute Walk Test in People With Stroke. *Journal of Neurologic Physical Therapy: JNPT*.

[43] Xie L, Yoon BH, Park C, You JSH (2022). Optimal Intervention Timing for Robotic-Assisted Gait Training in Hemiplegic Stroke. *Brain Sciences*.

[44] Arboix A, Massons J, García-Eroles L, Targa C, Comes E, Parra O (2010). Nineteen-year trends in risk factors, clinical characteristics and prognosis in lacunar infarcts. *Neuroepidemiology*.

[45] Torres-Riera S, Arboix A, Parra O, García-Eroles L, Sánchez-López MJ (2025). Predictive Clinical Factors of In-Hospital Mortality in Women Aged 85 Years or More with Acute Ischemic Stroke. *Cerebrovascular Diseases (Basel, Switzerland)*.

[46] Pujadas Capmany R, Arboix A, Casañas-Muñoz R, Anguera-Ferrando N (2004). Specific cardiac disorders in 402 consecutive patients with ischaemic cardioembolic stroke. *International Journal of Cardiology*.

