# Supplementary material for: Gait Training Using Robotic Devices in Subjects With Stroke: An Overview of Systematic Reviews and Meta-analysis
Source: Rev Neurol. 2026 Mar 18;81(3):46880. [Article in Spanish] doi: 10.31083/RN46880 (PMC13036787; doi:10.31083/RN46880)
Supplement: Supplementary file 1 [file 1576-6578-81-3-46880-s1.zip › PRISMA-Checklist.pdf]

Tabla 1. Lista de verificación PRISMA 2020

Sección/Tema	Ítem n.º	Ítem de la lista de verificación	Localización del ítem en el reporte
**TÍTULO**			
Título	1	Identifique el reporte como una revisión sistemática.	1
**RESUMEN**			
Resumen estructurado	2	Vea la lista de verificación para resúmenes estructurados de la declaración PRISMA 2020 (Tabla 2).	1
**INTRODUCCIÓN**			
Justificación	3	Describa la justificación de la revisión en el contexto del conocimiento existente.	2
Objetivos	4	Proporcione una declaración explícita de los objetivos o las preguntas que aborda la revisión.	2
**MÉTODOS**			
Criterios de elegibilidad	5	Especifique los criterios de inclusión y exclusión de la revisión y cómo se agruparon los estudios para la síntesis.	2
Fuentes de información	6	Especifique todas las bases de datos, registros, sitios web, organizaciones, listas de referencias y otros recursos de búsqueda o consulta para identificar los estudios. Especifique la fecha en la que cada recurso se buscó o consultó por última vez.	3
Estrategia de búsqueda	7	Presente las estrategias de búsqueda completas de todas las bases de datos, registros y sitios web, incluyendo cualquier filtro y los límites utilizados.	3
Proceso de selección de los estudios	8	Especifique los métodos utilizados para decidir si un estudio cumple con los criterios de inclusión de la revisión, incluyendo cuántos autores de la revisión cribaron cada registro y cada reporte recuperado, si trabajaron de manera independiente y, si procede, los detalles de las herramientas de automatización utilizadas en el proceso.	3
Proceso de extracción de los datos	9	Indique los métodos utilizados para extraer los datos de los informes o reportes, incluyendo cuántos revisores recopilaron datos de cada reporte, si trabajaron de manera independiente, los procesos para obtener o confirmar los datos por parte de los investigadores del estudio y, si procede, los detalles de las herramientas de automatización utilizadas en el proceso.	3

Sección/Tema	Ítem n.º	Ítem de la lista de verificación	Localización del ítem en el reporte
Lista de los datos	10a	Enumere y defina todos los desenlaces para los que se buscaron los datos. Especifique si se buscaron todos los resultados compatibles con cada dominio del desenlace (por ejemplo, para todas las escalas de medida, puntos temporales, análisis) y, de no ser así, los métodos utilizados para decidir los resultados que se debían recoger.	3
	10b	Enumere y defina todas las demás variables para las que se buscaron datos (por ejemplo, características de los participantes y de la intervención, fuentes de financiación). Describa todos los supuestos formulados sobre cualquier información ausente (*missing*) o incierta.	3
Evaluación del riesgo de sesgo de los estudios individuales	11	Especifique los métodos utilizados para evaluar el riesgo de sesgo de los estudios incluidos, incluyendo detalles de las herramientas utilizadas, cuántos autores de la revisión evaluaron cada estudio y si trabajaron de manera independiente y, si procede, los detalles de las herramientas de automatización utilizadas en el proceso.	3
Medidas del efecto	12	Especifique, para cada desenlace, las medidas del efecto (por ejemplo, razón de riesgos, diferencia de medias) utilizadas en la síntesis o presentación de los resultados.	3
Métodos de síntesis	13a	Describa el proceso utilizado para decidir qué estudios eran elegibles para cada síntesis (por ejemplo, tabulando las características de los estudios de intervención y comparándolas con los grupos previstos para cada síntesis (ítem n.º 5).	3
	13b	Describa cualquier método requerido para preparar los datos para su presentación o síntesis, tales como el manejo de los datos perdidos en los estadísticos de resumen o las conversiones de datos.	3
	13c	Describa los métodos utilizados para tabular o presentar visualmente los resultados de los estudios individuales y su síntesis.	3
	13d	Describa los métodos utilizados para sintetizar los resultados y justifique sus elecciones. Si se ha realizado un metaanálisis, describa los modelos, los métodos para identificar la presencia y el alcance de la heterogeneidad estadística, y los programas informáticos utilizados.	3
	13e	Describa los métodos utilizados para explorar las posibles causas de heterogeneidad entre los resultados de los estudios (por ejemplo, análisis de subgrupos, metarregresión).	3

Sección/Tema	Ítem n.º	Ítem de la lista de verificación	Localización del ítem en el reporte
	13f	Describa los análisis de sensibilidad que se hayan realizado para evaluar la robustez de los resultados de la síntesis.	-
Evaluación del sesgo en el reporte	14	Describa los métodos utilizados para evaluar el riesgo de sesgo debido a resultados faltantes en una síntesis (derivados de los sesgos en los reportes).	3
Evaluación de la certeza de la evidencia	15	Describa los métodos utilizados para evaluar la certeza (o confianza) en el cuerpo de la evidencia para cada desenlace.	3
**RESULTADOS**			
Selección de los estudios	16a	Describa los resultados de los procesos de búsqueda y selección, desde el número de registros identificados en la búsqueda hasta el número de estudios incluidos en la revisión, idealmente utilizando un diagrama de flujo (ver Figura 1).	3
	16b	Cite los estudios que aparentemente cumplían con los criterios de inclusión, pero que fueron excluidos, y explique por qué fueron excluidos.	3
Características de los estudios	17	Cite cada estudio incluido y presente sus características.	3,4
Riesgo de sesgo de los estudios individuales	18	Presente las evaluaciones del riesgo de sesgo para cada uno de los estudios incluidos.	5
Resultados de los estudios individuales	19	Presente, para todos los desenlaces y para cada estudio: a) los estadísticos de resumen para cada grupo (si procede) y b) la estimación del efecto y su precisión (por ejemplo, intervalo de credibilidad o de confianza), idealmente utilizando tablas estructuradas o gráficos.	6,7,8,9,10
Resultados de la síntesis	20a	Para cada síntesis, resuma brevemente las características y el riesgo de sesgo entre los estudios contribuyentes.	6,7,8,9,10
	20b	Presente los resultados de todas las síntesis estadísticas realizadas. Si se ha realizado un metaanálisis, presente para cada uno de ellos el estimador de resumen y su precisión (por	6,7,8,9,10

Sección/Tema	Ítem n.º	Ítem de la lista de verificación	Localización del ítem en el reporte
		ejemplo, intervalo de credibilidad o de confianza) y las medidas de heterogeneidad estadística. Si se comparan grupos, describa la dirección del efecto.	
	20c	Presente los resultados de todas las investigaciones sobre las posibles causas de heterogeneidad entre los resultados de los estudios.	6, 7, 8, 9, 10
	20d	Presente los resultados de todos los análisis de sensibilidad realizados para evaluar la robustez de los resultados sintetizados.	-
Sesgos en el reporte	21	Presente las evaluaciones del riesgo de sesgo debido a resultados faltantes (derivados de los sesgos del reporte) para cada síntesis evaluada.	6, 7, 8, 9, 10
Certeza de la evidencia	22	Presente las evaluaciones de la certeza (o confianza) en el cuerpo de la evidencia para cada desenlace evaluado.	6, 7, 8, 9, 10
**DISCUSIÓN**			
Discusión	23a	Proporcione una interpretación general de los resultados en el contexto de otras evidencias.	12,15
	23b	Argumente las limitaciones de la evidencia incluida en la revisión.	15
	23c	Argumente las limitaciones de los procesos de revisión utilizados.	15
	23d	Argumente las implicaciones de los resultados para la práctica, las políticas y las futuras investigaciones.	15
**OTRA INFORMACIÓN**			
Registro y protocolo	24a	Proporcione la información del registro de la revisión, incluyendo el nombre y el número de registro, o declare que la revisión no ha sido registrada.	1
	24b	Indique dónde se puede acceder al protocolo, o declare que no se ha redactado ningún protocolo.	1
	24c	Describa y explique cualquier enmienda a la información proporcionada en el registro o en el protocolo.	-
Financiación	25	Describa las fuentes de apoyo financiero o no financiero para la revisión y el papel de los financiadores o patrocinadores en la revisión.	16

Sección/Tema	Ítem n.º	Ítem de la lista de verificación	Localización del ítem en el reporte
Conflicto de intereses	26	Declare los conflictos de intereses de los autores de la revisión.	16
Disponibilidad de datos, códigos y otros materiales	27	Especifique qué elementos de los que se indican a continuación están disponibles al público y dónde se pueden encontrar: plantillas de formularios de extracción de datos, datos extraídos de los estudios incluidos, datos utilizados para todos los análisis, código de análisis, cualquier otro material utilizado en la revisión.	16

Tabla 2. Lista de verificación PRISMA 2020 para resúmenes estructurados*

Sección/Tema	Ítem n.º	Ítem de la lista de verificación
**TÍTULO**		
Título	1	Identifique el informe o reporte como una revisión sistemática.
**ANTECEDENTES**		
Objetivos	2	Proporcione una declaración explícita de los principales objetivos o preguntas que aborda la revisión.
**MÉTODOS**		
Criterios de elegibilidad	3	Especifique los criterios de inclusión y exclusión de la revisión.
Fuentes de información	4	Especifique las fuentes de información (por ejemplo, bases de datos, registros) utilizadas para identificar los estudios y la fecha de la última búsqueda en cada una de estas fuentes.
Riesgo de sesgo de los estudios individuales	5	Especifique los métodos utilizados para evaluar el riesgo de sesgo de los estudios individuales incluidos.
Síntesis de los resultados	6	Especifique los métodos utilizados para presentar y sintetizar los resultados.
**RESULTADOS**		
Estudios incluidos	7	Proporcione el número total de estudios incluidos y de participantes y resuma las características relevantes de los estudios.
Síntesis de los resultados	8	Presente los resultados de los desenlaces principales e indique, preferiblemente, el número de estudios incluidos y los participantes en cada uno de ellos. Si se ha realizado un metaanálisis, indique el estimador de resumen y el intervalo de confianza o de credibilidad. Si se comparan grupos, describa la dirección del efecto (por ejemplo, qué grupo se ha visto favorecido).
**DISCUSIÓN**		
Limitaciones de la evidencia	9	Proporcione un breve resumen de las limitaciones de la evidencia incluida en la revisión (por ejemplo, riesgo de sesgo, inconsistencia –heterogeneidad– e imprecisión).
Interpretación	10	Proporcione una interpretación general de los resultados y sus implicaciones importantes.
**OTROS**		
Financiación	11	Especifique la fuente principal de financiación de la revisión.
Registro	12	Proporcione el nombre y el número de registro.

*Esta lista de verificación conserva los mismos ítems que se incluyeron en la declaración PRISMA para resúmenes publicada en 2013 (48), pero ha sido revisada para que la redacción sea coherente con la declaración PRISMA 2020. Además, incluye un nuevo ítem que recomienda a los autores que especifiquen los métodos utilizados para presentar y sintetizar los resultados (ítem n.º 6).
