# Supplementary material for: Gait Training Using Robotic Devices in Subjects With Stroke: An Overview of Systematic Reviews and Meta-analysis
Source: Rev Neurol. 2026 Mar 18;81(3):46880. [Article in Spanish] doi: 10.31083/RN46880 (PMC13036787; doi:10.31083/RN46880)
Supplement: Supplementary file 1 [file 1576-6578-81-3-46880-s1.zip › Supplementary Material.docx]

**Suplementario Tabla 1.** Estrategia de búsqueda en base de datos Pubmed.

| **Paciente** | ("Stroke" OR "Cerebrovascular Accident" OR "CVA" OR "Ictus" OR "Cerebrovascular Stroke" OR "Brain Attack" OR "Brain Vascular Accident" OR "Cerebral Infarction" OR "Ischemic Stroke" OR "Hemorrhagic Stroke" OR "Thrombotic Stroke" OR "Embolic Stroke" OR "Transient Ischemic Attack" OR "TIA") |
| --- | --- |
| **Intervención** | ("Exoskeleton"[Title/Abstract] OR "Exoskeletons"[Title/Abstract] OR "Powered Exoskeleton"[Title/Abstract] OR "Robotic Exoskeleton” "[All Fields]) OR (“end-effector” [All fields]) OR |
| **Intervención en el grupo control** | #1 ("Control"[Title/Abstract] OR "Placebo"[Title/Abstract] OR "No Treatment"[Title/Abstract] OR "Standard Care"[Title/Abstract] OR "Conventional Therapy"[Title/Abstract] OR "Usual Care"[Title/Abstract] OR "Alternative Therapy"[Title/Abstract]) |
| **Desenlace o medida de resultado** | #1 "gait"[MeSH Terms] OR "gait"[All Fields] OR "gait apraxia"[MeSH Terms] OR "walk*"[All Fields] OR "ambulat*"[All Fields] OR "recovery of function"[All Fields]”  #2 "Gait Recovery"[All Fields] OR "Walking Recovery"[All Fields] OR "Ambulation Recovery"[All Fields] OR "Locomotion Recovery"[All Fields] OR "Gait Rehabilitation"[All Fields] OR "Walking Rehabilitation"[All Fields] OR "Ambulation Rehabilitation"[All Fields] OR "Locomotion Rehabilitation"[All Fields] OR "Gait Improvement"[All Fields] OR "Walking Improvement"[All Fields] OR "Ambulation Improvement"[All Fields] OR "Locomotion Improvement"[All Fields] OR "Timed Up and Go"[All Fields] OR "TUG test"[All Fields] OR "Get Up and Go test"[All Fields] OR ("walk test"[All Fields])) OR (("time"[MeSH Terms] OR "time"[All Fields]) AND ("walk test"[MeSH Terms] OR ("walk"[All Fields] AND "test"[All Fields]) OR "walk test"[All Fields])) OR "Gait Velocity"[All Fields] OR "Walking Speed"[All Fields] OR "Gait Speed"[All Fields] OR "Ambulation Speed"[All Fields] OR "Walking Velocity"[All Fields] OR "Locomotion Speed"[All Fields] OR "Stride Speed"[All Fields] OR "10-Meter Walk Test"[All Fields] OR "10MWT"[All Fields] OR "Ten Meter Walk Test"[All Fields] OR "10 Meter Walking Test"[All Fields] OR ("10"[All Fields] AND ("meter"[All Fields] OR "meter s"[All Fields]) AND ("gait"[MeSH Terms] OR "gait"[All Fields]) OR "Fugl-Meyer"[All Fields] OR "Fugl-Meyer Assessment"[All Fields] OR "Fugl-Meyer Motor Scale"[All Fields] OR "Fugl-Meyer Evaluation"[All Fields] OR "Fugl-Meyer Score"[All Fields] OR "Fugl-Meyer Test"[All Fields] OR "Tinetti"[All Fields] OR "Tinetti Test"[All Fields] OR "Tinetti Scale"[All Fields] OR "Tinetti Assessment"[All Fields] OR "POMA"[All Fields] OR "Tinetti Balance Test"[All Fields] OR "Spatio-Temporal Parameters"[All Fields] OR "Gait Parameters"[All Fields] OR "Walking Parameters"[All Fields] OR "Stride Parameters"[All Fields] OR "Temporal-Spatial Gait Characteristics"[All Fields] OR "Kinematic Gait Analysis"[All Fields] OR "Gait Metrics"[All Fields] OR ("step"[All Fields] AND ("length"[All Fields] OR "lengths"[All Fields]) AND ("time"[MeSH Terms] OR "time"[All Fields])) OR (("stride"[All Fields] OR "stride s"[All Fields] OR "strides"[All Fields] OR "striding"[All Fields]) AND ("length"[All Fields] OR "lengths"[All Fields]) AND ("duration"[All Fields] OR "durations"[All Fields])) OR (("gait"[MeSH Terms] OR "gait"[All Fields]) |

**Suplementario Tabla 2.** Revisiones sistemáticas y metaanálisis excluidos y sus razones sobre el uso de dispositivos robóticos para el entrenamiento de la marcha en el ictus.

| **Artículos excluidos** | **Razones** |
| --- | --- |
| Powered robotic exoskeletons in post-stroke rehabilitation of gait: a scoping review.  Louie DR, Eng JJ. | Scoping review |
| The effect of ‘device-in-charge’ versus ‘patient-in-charge’ support during robotic gait training on walking ability and balance in chronic stroke survivors: A systematic review.  Haarman JAM, Reenalda J, Buurke JH, van der Kooij H, Rietman JS. | Otro objetivo de estudio |
| Wearable ankle robots in post-stroke rehabilitation of gait: a systematic review.  Shi B, Chen X, Yue Z, Yin S, Weng Q, Zhang X, Wang J, Wen W. | Falta de medidas para la marcha |
| Systematic review on wearable lower‑limb exoskeletons for gait training in neuromuscular impairments.  Rodríguez-Fernández A, Lobo-Prat J, Font-Llagunes JM. | Incluye estudios observacionales y pilotos, pocos ECA. Estudio descriptivo de férulas robóticas. |
| Effects of gait training with the Hybrid Assistive Limb on gait ability in stroke patients: a systematic review of randomized controlled trials.  Taki S, Iwamoto Y, Imura T, Mitsutake T, Tanaka R. | *Falta de estudios incluidos* |
| Effectiveness of robo-assisted lower limb rehabilitation for spastic patients: a systematic review.  Shakti D, Mathew L, Kumar N, Kataria C. | Sin acceso a los datos |

**Suplementario Tabla 3.** Resumen de los ensayos clínicos incluidos en las revisiones sistemáticas y metaanálisis sobre el uso de dispositivos robóticos para el

entrenamiento de la marcha en el ictus.

| **ARTÍCULOS** | Cho et al. (2018) (73) | Bruni et al. (2018) (134) | Postol et al. (2019) (80) | Tedla et al. (2019) (74) | Maranesi et al. (2019) (133) | Schröder et al. (2019) (132) | Mehrholz et al. (2020) (20) | Moucheboeuf et al. (2020) (63) | Hsu et al. (2020) (131) | Nedergard et al. (2021) (130) | Calafiore et al. (2021) (128) | Hsu et al. (2022) (129) | Zhang et al. (2023) (127) |
| --- | --- | --- | --- | --- | --- | --- | --- | --- | --- | --- | --- | --- | --- |
| Ada 2010 |  |  |  |  |  |  |  |  |  |  |  |  |  |
| Aleksandra S. Dragin 2014 |  |  |  |  |  |  |  |  |  |  |  |  |  |
| Aprile 2017 |  |  |  |  |  |  |  |  |  |  |  |  |  |
| Aprile 2019 |  |  |  |  |  |  |  |  |  |  |  |  |  |
| Aschbacher 2006 |  |  |  |  |  |  |  |  |  |  |  |  |  |
| Bang 2016 |  |  |  |  |  |  |  |  |  |  |  |  |  |
| Belas Dos Santos 2018 |  |  |  |  |  |  |  |  |  |  |  |  |  |
| Bergmann 2018 |  |  |  |  |  |  |  |  |  |  |  |  |  |
| Bojek 2019 |  |  |  |  |  |  |  |  |  |  |  |  |  |
| Brincks 2011 |  |  |  |  |  |  |  |  |  |  |  |  |  |
| Buesing 2015 |  |  |  |  |  |  |  |  |  |  |  |  |  |
| Calabró 2018 |  |  |  |  |  |  |  |  |  |  |  |  |  |
| Chang 2012 |  |  |  |  |  |  |  |  |  |  |  |  |  |
| Chua 2016 |  |  |  |  |  |  |  |  |  |  |  |  |  |
| Dae-Hypuk Bang 2016 |  |  |  |  |  |  |  |  |  |  |  |  |  |
| Dean 2010 |  |  |  |  |  |  |  |  |  |  |  |  |  |
| DeLuca 2020 |  |  |  |  |  |  |  |  |  |  |  |  |  |
| Días 2007 |  |  |  |  |  |  |  |  |  |  |  |  |  |
| Dong-Hoon Kim 2021 |  |  |  |  |  |  |  |  |  |  |  |  |  |
| Dong-Xia Li 2021 |  |  |  |  |  |  |  |  |  |  |  |  |  |
| Dragin 2014 |  |  |  |  |  |  |  |  |  |  |  |  |  |
| Eun Young Han 2016 |  |  |  |  |  |  |  |  |  |  |  |  |  |
| Fisher 2008 |  |  |  |  |  |  |  |  |  |  |  |  |  |
| Fisher 2011 |  |  |  |  |  |  |  |  |  |  |  |  |  |
| Forrester 2014 |  |  |  |  |  |  |  |  |  |  |  |  |  |
| Franceschini 2009 |  |  |  |  |  |  |  |  |  |  |  |  |  |
| Gandolfi 2019 |  |  |  |  |  |  |  |  |  |  |  |  |  |
| Genna Waldman 2013 |  |  |  |  |  |  |  |  |  |  |  |  |  |
| Geroin 2011 |  |  |  |  |  |  |  |  |  |  |  |  |  |
| Giovanni Taveggia 2016 |  |  |  |  |  |  |  |  |  |  |  |  |  |
| Han 2016 |  |  |  |  |  |  |  |  |  |  |  |  |  |
| Hayeon Kim 2020 |  |  |  |  |  |  |  |  |  |  |  |  |  |
| Hesse 2012 |  |  |  |  |  |  |  |  |  |  |  |  |  |
| Hidler 2009 |  |  |  |  |  |  |  |  |  |  |  |  |  |
| Hiroki Watanabe 2016 |  |  |  |  |  |  |  |  |  |  |  |  |  |
| Hornby 2008 |  |  |  |  |  |  |  |  |  |  |  |  |  |
| Husemann 2007 |  |  |  |  |  |  |  |  |  |  |  |  |  |
| Jayaraman 2019 |  |  |  |  |  |  |  |  |  |  |  |  |  |
| Karla Bustamante Valles 2016 |  |  |  |  |  |  |  |  |  |  |  |  |  |
| Kelley 2013 |  |  |  |  |  |  |  |  |  |  |  |  |  |
| Kim 2019a |  |  |  |  |  |  |  |  |  |  |  |  |  |
| Kim 2019b |  |  |  |  |  |  |  |  |  |  |  |  |  |
| Kimet 2018 |  |  |  |  |  |  |  |  |  |  |  |  |  |
| Kwan-Shan Chan 2012 |  |  |  |  |  |  |  |  |  |  |  |  |  |
| Kwon 2018 |  |  |  |  |  |  |  |  |  |  |  |  |  |
| Kyung 2008 |  |  |  |  |  |  |  |  |  |  |  |  |  |
| Larry W. Forrester 2014 |  |  |  |  |  |  |  |  |  |  |  |  |  |
| Lee 2019 |  |  |  |  |  |  |  |  |  |  |  |  |  |
| Lewek 2009 |  |  |  |  |  |  |  |  |  |  |  |  |  |
| Li 2021 |  |  |  |  |  |  |  |  |  |  |  |  |  |
| Ling‑Fung Yeung 202 |  |  |  |  |  |  |  |  |  |  |  |  |  |
| Mayr 2008 |  |  |  |  |  |  |  |  |  |  |  |  |  |
| Mayr 2018 |  |  |  |  |  |  |  |  |  |  |  |  |  |
| Michiel P. M. van Nunen 2014 |  |  |  |  |  |  |  |  |  |  |  |  |  |
| Molteni 2021 |  |  |  |  |  |  |  |  |  |  |  |  |  |
| Morone 2011 |  |  |  |  |  |  |  |  |  |  |  |  |  |
| Morone 2012 |  |  |  |  |  |  |  |  |  |  |  |  |  |
| Morone 2018 |  |  |  |  |  |  |  |  |  |  |  |  |  |
| Nam 2019 |  |  |  |  |  |  |  |  |  |  |  |  |  |
| Nam 2020 |  |  |  |  |  |  |  |  |  |  |  |  |  |
| Natapatchakrid Thimabut 2022 |  |  |  |  |  |  |  |  |  |  |  |  |  |
| Ng 2008 |  |  |  |  |  |  |  |  |  |  |  |  |  |
| Nilsson 2001 |  |  |  |  |  |  |  |  |  |  |  |  |  |
| Noser 2012 |  |  |  |  |  |  |  |  |  |  |  |  |  |
| Ochi 2015 |  |  |  |  |  |  |  |  |  |  |  |  |  |
| Ogino 2020 |  |  |  |  |  |  |  |  |  |  |  |  |  |
| Park 2018 |  |  |  |  |  |  |  |  |  |  |  |  |  |
| Peurala 2005 |  |  |  |  |  |  |  |  |  |  |  |  |  |
| Peurala 2009 |  |  |  |  |  |  |  |  |  |  |  |  |  |
| Picelli 2016 |  |  |  |  |  |  |  |  |  |  |  |  |  |
| Pohl 2007 |  |  |  |  |  |  |  |  |  |  |  |  |  |
| Pohl 2017 |  |  |  |  |  |  |  |  |  |  |  |  |  |
| Rojek 2019 |  |  |  |  |  |  |  |  |  |  |  |  |  |
| Rosaria De Luca 2020 |  |  |  |  |  |  |  |  |  |  |  |  |  |
| Rustem Mustafaoglu 2020 |  |  |  |  |  |  |  |  |  |  |  |  |  |
| Saltuari 2004 |  |  |  |  |  |  |  |  |  |  |  |  |  |
| Schwartz 2009 |  |  |  |  |  |  |  |  |  |  |  |  |  |
| Sczesny-Kaiser 2019 |  |  |  |  |  |  |  |  |  |  |  |  |  |
| Srivastava 2016 |  |  |  |  |  |  |  |  |  |  |  |  |  |
| Stein 2014 |  |  |  |  |  |  |  |  |  |  |  |  |  |
| Stolz 2019 |  |  |  |  |  |  |  |  |  |  |  |  |  |
| Suat Erel 2011 |  |  |  |  |  |  |  |  |  |  |  |  |  |
| Tanaka 2012 |  |  |  |  |  |  |  |  |  |  |  |  |  |
| Tanaka 2019 |  |  |  |  |  |  |  |  |  |  |  |  |  |
| Taveggia 2016 |  |  |  |  |  |  |  |  |  |  |  |  |  |
| Tomida 2019 |  |  |  |  |  |  |  |  |  |  |  |  |  |
| Tomoyuki Ogino 2020 |  |  |  |  |  |  |  |  |  |  |  |  |  |
| Tong 2006 |  |  |  |  |  |  |  |  |  |  |  |  |  |
| Ucar 2014 |  |  |  |  |  |  |  |  |  |  |  |  |  |
| Van numen 2012 |  |  |  |  |  |  |  |  |  |  |  |  |  |
| Van nunen 2015 |  |  |  |  |  |  |  |  |  |  |  |  |  |
| Watanabe 2014 |  |  |  |  |  |  |  |  |  |  |  |  |  |
| Watanabe 2017 |  |  |  |  |  |  |  |  |  |  |  |  |  |
| Werner 2002 |  |  |  |  |  |  |  |  |  |  |  |  |  |
| Westlake 2009 |  |  |  |  |  |  |  |  |  |  |  |  |  |
| Wonho Choi 2022 |  |  |  |  |  |  |  |  |  |  |  |  |  |
| Wright 2020 |  |  |  |  |  |  |  |  |  |  |  |  |  |
| Wu 2012 |  |  |  |  |  |  |  |  |  |  |  |  |  |
| Yeung 2018 |  |  |  |  |  |  |  |  |  |  |  |  |  |
| Yoshikawa 2017 |  |  |  |  |  |  |  |  |  |  |  |  |  |
| Yun 2019 |  |  |  |  |  |  |  |  |  |  |  |  |  |

**Suplementario Tabla 4.** Tabla resumen de la certeza de la evidencia según GRADE por medida de resultado sobre el uso de dispositivos robóticos para el entrenamiento de la marcha en el ictus.

**Autor(es):** Mehrholz et al. ^32^

**Pregunta:** Un dispositivo robótico de asistencia a la marcha en combinación con fisioterapia comparado con fisioterapia convencional para mejorar la marcha en pacientes post ictus

| **Evaluación de certeza** | | | | | | | **№ de pacientes** | | **Efecto** | | **Certeza** | **Importancia** |
| --- | --- | --- | --- | --- | --- | --- | --- | --- | --- | --- | --- | --- |
| **№ de estudios** | **Diseño de estudio** | **Riesgo de sesgo** | **Inconsistencia** | **Evidencia indirecta** | **Imprecisión** | **Otras consideraciones** | **un dispositivo robótico de asistencia a la marcha en combinación con fisioterapia** | **fisioterapia convencional** | **Relativo (95% CI)** | **Absoluto (95% CI)** |  |  |
| **Velocidad de la marcha al final del tratamiento (evaluado con : 10MWT)** | | | | | | | | | | | | |
| 42 | ensayos aleatorios | serio^a^ | serio^b^ | no es serio | no es serio | ninguno | 836 | 764 | - | MD **0.06 m/s más alto.** (0.02 más alto. a 0.1 más alto.) | ⨁⨁◯◯ Baja^a,b^ | CRÍTICO |
| **Velocidad de la marcha durante el seguimiento (evaluado con : 10MWT)** | | | | | | | | | | | | |
| 13 | ensayos aleatorios | serio^a^ | serio^c^ | no es serio | no es serio | ninguno | 377 | 350 | - | MD **0.07 m/s más alto.** (0.03 menor a 0.17 más alto.) | ⨁⨁◯◯ Baja^a,c^ | CRÍTICO |
| **Movilidad funcional (evaluado con : FAC)** | | | | | | | | | | | | |
| 38 | ensayos aleatorios | serio^a^ | no es serio | no es serio | no es serio | fuerte asociación ^d^ | 440/806 (54.6%) | 339/761 (44.5%) | **OR 2.14** (1.57 a 2.92) | **187 más por 1000** (de 112 más a 256 más ) | ⨁⨁⨁⨁ Alta^a,d^ | CRÍTICO |
| **Movilidad funcional durante el seguimiento (seguimiento: media 22.3 semanas; evaluado con : FAC)** | | | | | | | | | | | | |
| 6 | ensayos aleatorios | serio^a^ | serio^e^ | no es serio | serio^f,g^ | ninguno | 172/249 (69.1%) | 136/247 (55.1%) | **OR 1.93** (0.72 a 5.13) | **152 más por 1000** (de 82 menos a 312 más ) | ⨁◯◯◯ Muy baja^a,e,f,g^ | CRÍTICO |
| **Resistencia (evaluado con : 6MWT)** | | | | | | | | | | | | |
| 24 | ensayos aleatorios | serio^a^ | serio^h^ | no es serio | serio^f,g^ | ninguno | 507 | 476 | - | MD **10.86 metros más alto.** (5.72 menor a 27.44 más alto.) | ⨁◯◯◯ Muy baja^a,f,g,h^ | IMPORTANTE |
| **Resistencia durante el seguimiento (evaluado con : 6MWT)** | | | | | | | | | | | | |
| 11 | ensayos aleatorios | serio^a^ | serio^b^ | no es serio | serio^f,g^ | ninguno | 313 | 299 | - | MD **7.76 metros más alto.** (21.47 menor a 36.99 más alto.) | ⨁◯◯◯ Muy baja^a,b,f,g^ | IMPORTANTE |

**CI:** Intervalo de confianza; **MD:** Diferencia media; **OR:** Razón de momios

Explicaciones

a. Estudios con riesgo de sesgos en cegamientos del evaluador.

b. Inconsistencia moderada del 60% con variaciones en el efecto en favor y en contra del tratamiento.

c. Inconsistencia alta del 74% con variaciones en el efecto en favor y en contra del tratamiento.

d. Aumento un nivel por efecto grande en la intervención (OR>2)

e. Inconsistencia alta del 79%

f. El IC cruza el umbral de decisión clínica entre recomendar y no recomendar el tratamiento.

g. El IC es demasiado amplio e impreciso

h. Inconsistencia moderada del 42%

**Autor(es):** Mehrholz et al. ^32^

**Pregunta:** Exoesqueleto comparado con terapia convencional para mejorar la marcha en pacientes post ictus

| **Evaluación de certeza** | | | | | | | **№ de pacientes** | | **Efecto** | | **Certeza** | **Importancia** |
| --- | --- | --- | --- | --- | --- | --- | --- | --- | --- | --- | --- | --- |
| **№ de estudios** | **Diseño de estudio** | **Riesgo de sesgo** | **Inconsistencia** | **Evidencia indirecta** | **Imprecisión** | **Otras consideraciones** | **exoesqueleto** | **terapia convencional** | **Relativo (95% CI)** | **Absoluto (95% CI)** |  |  |
| **Velocidad de la marcha (evaluado con : m/s)** | | | | | | | | | | | | |
| 23 | ensayos aleatorios | no es serio | no es serio | no es serio | no es serio | ninguno | 379 | 363 | - | MD **0**  (0.05 menor a 0.04 más alto.) | ⨁⨁⨁⨁ Alta | CRÍTICO |
| **Movilidad funcional (evaluado con : FAC)** | | | | | | | | | | | | |
| 18 | ensayos aleatorios | no es serio | no es serio | no es serio | no es serio | ninguno | 156/349 (44.7%) | 194/336 (57.7%) | **OR 2.11** (1.36 a 3.29) | **165 más por 1000** (de 73 más a 241 más ) | ⨁⨁⨁⨁ Alta | CRÍTICO |

**CI:** Intervalo de confianza; **MD:** Diferencia media; **OR:** Razón de momios

**Autor(es):** Mehrholz et al. ^32^

**Pregunta:** Efector final comparado con terapia convencional para mejorar la marcha en pacientes post ictus

| **Evaluación de certeza** | | | | | | | **№ de pacientes** | | **Efecto** | | **Certeza** | **Importancia** |
| --- | --- | --- | --- | --- | --- | --- | --- | --- | --- | --- | --- | --- |
| **№ de estudios** | **Diseño de estudio** | **Riesgo de sesgo** | **Inconsistencia** | **Evidencia indirecta** | **Imprecisión** | **Otras consideraciones** | **efector final** | **terapia convencional** | **Relativo (95% CI)** | **Absoluto (95% CI)** |  |  |
| **Velocidad (evaluado con : m/s)** | | | | | | | | | | | | |
| 13 | ensayos aleatorios | no es serio | serio^a^ | no es serio | no es serio | ninguno | 360 | 305 | - | MD **0.12 más alto.** (0.05 más alto. a 0.19 más alto.) | ⨁⨁⨁◯ Moderado^a^ | CRÍTICO |
| **Movilidad funcional (evaluado con : FAC)** | | | | | | | | | | | | |
| 11 | ensayos aleatorios | no es serio | no es serio | no es serio | no es serio | ninguno | 159/312 (51.0%) | 102/286 (35.7%) | **OR 1.90** (0.99 a 3.63) | **156 más por 1000** (de 2 menos a 311 más ) | ⨁⨁⨁⨁ Alta | CRÍTICO |

**CI:** Intervalo de confianza; **MD:** Diferencia media; **OR:** Razón de momios

Explicaciones

a. Heterogeneidad 69%

**Autor(es):** Mehrholz et al. ^32^

**Pregunta:** Dispositivo robótico en pacientes en fase aguda comparado con tratamiento convencional para mejorar la marcha en pacientes post ictus

| **Evaluación de certeza** | | | | | | | **№ de pacientes** | | **Efecto** | | **Certeza** | **Importancia** |
| --- | --- | --- | --- | --- | --- | --- | --- | --- | --- | --- | --- | --- |
| **№ de estudios** | **Diseño de estudio** | **Riesgo de sesgo** | **Inconsistencia** | **Evidencia indirecta** | **Imprecisión** | **Otras consideraciones** | **dispositivo robótico en pacientes en fase aguda** | **tratamiento convencional** | **Relativo (95% CI)** | **Absoluto (95% CI)** |  |  |
| **Movilidad funcional (evaluado con : FAC)** | | | | | | | | | | | | |
| 24 | ensayos aleatorios | no es serio | no es serio | no es serio | no es serio | ninguno | 243/629 (38.6%) | 169/614 (27.5%) | **OR 1.96** (1.47 a 2.62) | **151 más por 1000** (de 83 más a 224 más ) | ⨁⨁⨁⨁ Alta | CRÍTICO |

**CI:** Intervalo de confianza; **OR:** Razón de momios

**Autor(es):** Mehrholz et al. ^32^

**Pregunta:** Dispositivo robótico en pacientes crónicos comparado con tratamiento convencional para mejorar la marcha en pacientes post ictus

| **Evaluación de certeza** | | | | | | | **№ de pacientes** | | **Efecto** | | **Certeza** | **Importancia** |
| --- | --- | --- | --- | --- | --- | --- | --- | --- | --- | --- | --- | --- |
| **№ de estudios** | **Diseño de estudio** | **Riesgo de sesgo** | **Inconsistencia** | **Evidencia indirecta** | **Imprecisión** | **Otras consideraciones** | **dispositivo robótico en pacientes crónicos** | **tratamiento convencional** | **Relativo (95% CI)** | **Absoluto (95% CI)** |  |  |
| **Movilidad funcional (evaluado con : FAC)** | | | | | | | | | | | | |
| 16 | ensayos aleatorios | no es serio | no es serio | no es serio | no es serio | ninguno | 212/248 (85.5%) | 189/213 (88.7%) | **OR 1.20** (0.40 a 3.65) | **17 más por 1000** (de 128 menos a 79 más ) | ⨁⨁⨁⨁ Alta | CRÍTICO |

**CI:** Intervalo de confianza; **OR:** Razón de momios

**Autor(es):** Mehrholz et al. ^32^

**Pregunta:** Dispositivo robótico en pacientes independientes comparado con terapia convencional para mejorar la marcha en pacientes post ictus

| **Evaluación de certeza** | | | | | | | **№ de pacientes** | | **Efecto** | | **Certeza** | **Importancia** |
| --- | --- | --- | --- | --- | --- | --- | --- | --- | --- | --- | --- | --- |
| **№ de estudios** | **Diseño de estudio** | **Riesgo de sesgo** | **Inconsistencia** | **Evidencia indirecta** | **Imprecisión** | **Otras consideraciones** | **dispositivo robótico en pacientes independientes** | **terapia convencional** | **Relativo (95% CI)** | **Absoluto (95% CI)** |  |  |
| **Velocidad de la marcha (evaluado con : m/s)** | | | | | | | | | | | | |
| 22 | ensayos aleatorios | no es serio | serio^a^ | no es serio | no es serio | ninguno | 377 | 338 | - | MD **0.02 más alto.** (0.04 menor a 0.09 más alto.) | ⨁⨁⨁◯ Moderado^a^ | CRÍTICO |

**CI:** Intervalo de confianza; **MD:** Diferencia media

Explicaciones

a. Heterogeneidad del 56%

**Autor(es):** Mehrholz et al. ^32^

**Pregunta:** Dispositivo robótico en pacientes dependientes comparado con terapia convencional para mejorar la marcha en pacientes post ictus

| **Evaluación de certeza** | | | | | | | **№ de pacientes** | | **Efecto** | | **Certeza** | **Importancia** |
| --- | --- | --- | --- | --- | --- | --- | --- | --- | --- | --- | --- | --- |
| **№ de estudios** | **Diseño de estudio** | **Riesgo de sesgo** | **Inconsistencia** | **Evidencia indirecta** | **Imprecisión** | **Otras consideraciones** | **dispositivo robótico en pacientes dependientes** | **terapia convencional** | **Relativo (95% CI)** | **Absoluto (95% CI)** |  |  |
| **Velocidad de la marcha (evaluado con : m/s)** | | | | | | | | | | | | |
| 11 | ensayos aleatorios | no es serio | serio^a^ | no es serio | no es serio | ninguno | 303 | 288 | - | MD **0.09 más alto.** (0.02 más alto. a 0.15 más alto.) | ⨁⨁⨁◯ Moderado^a^ | CRÍTICO |
| **Movilidad funcional (evaluado con : FAC)** | | | | | | | | | | | | |
| 14 | ensayos aleatorios | no es serio | no es serio | no es serio | no es serio | ninguno | 131/378 (34.7%) | 81/354 (22.9%) | **OR 2.02** (1.27 a 3.22) | **146 más por 1000** (de 45 más a 260 más ) | ⨁⨁⨁⨁ Alta | CRÍTICO |

**CI:** Intervalo de confianza; **MD:** Diferencia media; **OR:** Razón de momios

Explicaciones

a. Heterogeneidad 67%

**Autor(es):** Schroder et al. ^29^

**Pregunta:** Entrenamiento de marcha repetido comparado con fisioterapia convencional para mejorar la marcha en pacientes post ictus

| **Evaluación de certeza** | | | | | | | **№ de pacientes** | | **Efecto** | | **Certeza** | **Importancia** |
| --- | --- | --- | --- | --- | --- | --- | --- | --- | --- | --- | --- | --- |
| **№ de estudios** | **Diseño de estudio** | **Riesgo de sesgo** | **Inconsistencia** | **Evidencia indirecta** | **Imprecisión** | **Otras consideraciones** | **entrenamiento de marcha repetido** | **fisioterapia convencional** | **Relativo (95% CI)** | **Absoluto (95% CI)** |  |  |
| **Función motora (evaluado con : FMA)** | | | | | | | | | | | | |
| 4 | ensayos aleatorios | serio^a^ | no es serio | no es serio | muy serio ^b,d^ | ninguno | 91 | 88 | - | MD **0.52 más alto.** (1.54 menor a 2.59 más alto.) | ⨁◯◯◯ Muy baja^a,b,d^ | IMPORTANTE |
| **Fuerza muscular (evaluado con : MI-LE)** | | | | | | | | | | | | |
| 5 | ensayos aleatorios | serio^a^ | no es serio | no es serio | serio^b,c^ | ninguno | 364 | 187 | - | MD **3.64 más alto.** (2.88 menor a 10.57 más alto.) | ⨁⨁◯◯ Baja^a,b,c^ | IMPORTANTE |

**CI:** Intervalo de confianza; **MD:** Diferencia media; **OR:** Razón de momios

Explicaciones

a. Los estudios incluidos presentan riesgo de sesgo en cegamientos.

b. El IC sobrepasa el umbral entre recomendar y no un tratamiento.

c. El IC es demasiado amplio.

d. Bajo tamaño muestral (<400)
